# Comparative and phylogenetic analysis of complete chloroplast genomes of *Phrynium* s. s. and *Stachyphrynium* (Marantaceae) in China, including a new species

**DOI:** 10.3389/fpls.2025.1569683

**Published:** 2025-05-01

**Authors:** Zhiyi Lin, Zhichao Fan, Yaoqing Lan, Lin Fu, Feilong Xu, Yi Tong

**Affiliations:** ^1^ School of Pharmaceutical Science, Guangzhou University of Chinese Medicine, Guangzhou, China; ^2^ Key Laboratory of Plant Resources Conservation and Sustainable Utilization, South China Botanical Garden, Chinese Academy of Sciences, Guangzhou, China; ^3^ Information Department, The Second Affiliated Hospital of Guangzhou University of Chinese Medicine, Guangzhou, China

**Keywords:** *Phrynium*, *Stachyphrynium*, chloroplast genome, nuclear ribosomal DNA, phylogenomic analysis, *Phrynium pyramidale*

## Abstract

Plants of the genera *Phrynium* and *Stachyphrynium* traditionally used as ethnomedicine or for wrapping sticky rice dumpling in the tropical and south subtropical Asia, have a long history of ethnobotanical use. China represents the northernmost distribution of Marantaceae in Asia. Due to the notably similar leaf morphology between these genera, herbarium specimens are frequently misidentified, especially during the vegetative stages. Their morphological uniformity and unclear interspecific genetic relationships pose significant challenges to taxonomic classification and species identification. To date, systematic taxonomic revisions and phylogeny of their indigenous species remain lacking. In this study, we conducted comparative chloroplast genomes analyses of seven *Phrynium* and two *Stachyphrynium* species in China. The chloroplast genomes exhibited conserved structure, gene content, gene order and codon usage bias, but diverged in genomes size and the SC/IR boundaries. Four variable regions were identified as potential molecular markers for species identification. Phylogenetic analyses using CDS and nrDNA strongly support *Phrynium* and *Stachyphrynium* in China as two distinct monophyletic groups, with *Phrynium* subdivided into two clades. These findings advance our understanding of their molecular relationships and provide critical insights for identification, utilization, and conservation as medicinal plants. Finally, we describe and illustrate the new species *Phrynium pyramidale* Y. Tong & Z. Y. Lin.

## 1 Introduction

*Phrynium* Willd. and *Stachyphrynium* K. Schum. are members of the family Marantaceae (order Zingiberales), classified within the Commelinids clade of the monocotyledonous flowering plants according to APG IV system (The Angiosperm Phylogeny Group, 2016; Cole et al., 2019). Currently, *Phrynium* comprises approximately 20 species distributed across the tropical and some subtropical regions of Asia and Africa. In China, six species have been documented (Wu and Helen, 2000; Fang, 2002; Fu et al., 2017), with an additional new species described in this study. Both genera are widely used in traditional Chinese medicine. The herbs and roots are employed for their purported therapeutic effects in clearing heat, detoxifying, cooling the blood, and stopping bleeding, aiding in treating conditions such as hoarseness, sore throat and oral ulcer (Guangdong Institute of Botany, 1977; Wu, 1991; State Administration of Traditional Chinese Medicine, 1999; Guangxi Institute of Botany, 2016). Additionally, these plants are cultivated as ornamental plants in garden ditches and shaded areas (Cen et al., 2017). The leaves of *Phrynium* are traditionally used by Chinese ethnic groups to wrap sticky rice dumplings, owing to their natural preservative properties (Cen et al., 2017).

The genus *Phrynium* is characterized by several distinctive morphological features, including congested and capitate inflorescences, spirally arranged bracts, sepals longer than the corolla tubes, 3-locular ovary and dehiscent fruits (Wu and Helen, 2000; Clausager and Borchsenius, 2003; Suksthan et al., 2010). Seven *Phrynium* species are recorded in China, whereas only two *Stachyphrynium* species occur there. Morphologically, *Stachyphrynium* differs from *Phrynium* by its solitary spikes, two-ranked bracts and corolla tubes longer than the sepals (Wu and Helen, 2000; Clausager and Borchsenius, 2003). Since Willdenow (1797) established *Phrynium*, taxonomic studies have focused on clarifying generic boundaries within Marantaceae and intrageneric classification. Multiple analyses indicate that *Phrynium* is non-monophyletic, forming a clade with *Phacelophrynium*, *Monophrynium*, and *Cominsia* (Andersson, 2001; Prince and Kress, 2006; Suksathan et al., 2009; Ley and Claßen-Bockhoff, 2011). Consequently, Suksathan et al. (2009) merged these genera into an expanded *Phrynium* during their taxonomic revision of Asian Marantaceae. However, Asian Marantaceae remain understudied compared to South American counterparts. Existing works (e.g., Roxburgh, 1820; Ridley, 1899; Schumann, 1902; Gagnepain, 1932; Holttum, 1951; Clausager and Borchsenius, 2003) offer limited insights into species delimitation and distribution. No modern systematic revisions exist for these genera. Given the absence of *Phacelophrynium*, *Monophrynium*, and *Cominsia* in China, this study focuses on *Phrynium* sensu stricto (s.s.). Current classifications rely on floral and bract morphology (Clausager and Borchsenius, 2003; Suksthan et al., 2010; Niissalo et al., 2016). Leaf morphology is highly uniform, complicating pre-flowering identification. *Stachyphrynium placentarium* was historically placed in *Phrynium* due to its densely capitate inflorescence (Merrill, 1919; Clausager and Borchsenius, 2003). Notably, illustrations of *Phrynium rheedei* and *Stachyphrynium placentarium* in Chinese Materia Medica are mislabeled and should be interchanged (State Administration of Traditional Chinese Medicine, 1999). Herbarium specimens of *Phrynium* are frequently misidentified, underscoring the urgent need for a comprehensive taxonomic revision of *Phrynium* and *Stachyphrynium* in China. We recommend integrating phylogenomic approaches to resolve interspecific relationships and develop reliable molecular barcodes.

Phylogenomics, particularly through complete chloroplast genomes and nuclear genomes analysis, has proven effective in addressing phylogenetic challenges in taxonomically complex plant groups (Jansen et al., 2007; Moore et al., 2007, 2010). The chloroplast, a vital photosynthetic organelle, contains an independent genome typically structured as a double-stranded circular DNA molecule with a highly conserved quadripartite structure: a large single-copy region (LSC), a small single-copy region (SSC), and two inverted repeat regions (IRs). The contraction and expansion of the IR region contribute to the diversity of chloroplast genomes among different species (Aii et al., 1997; Plunkett and Downie, 2000; Wang et al., 2008). Most angiosperm chloroplast genomes range from 120 to 160 kb in length and exhibit low molecular weight, high copy number, structural conservation, maternal inheritance, and low recombination rate (Palmer et al., 1988; Green, 2011; Jansen and Ruhlman, 2012). Consequently, they are widely used in species identification, phylogenetics, and molecular evolution studies (Smith et al., 1993; Andersson, 2001; Prince and Kress, 2006; Suksathan et al., 2009). However, chloroplast genomes have limitations in resolving rapid radiation events and detecting hybridization or polyploidy (McKain et al., 2018). In contrast, nuclear genome possess key features such as biparental inheritance, abundant informative loci, and higher mutation rates (Kellogg and Bennetzen, 2004), enhancing their resolution for lower taxonomic ranks. Due to their large size and structural complexity, nuclear studies often focus on repetitive sequences (e.g., ribosomal DNA [rDNA]) or multigene families (Richard et al., 2008). Currently, rDNA is as pivotal as chloroplast genomes in plant systematics and taxonomy (Kress et al., 2001, 2002; Qiu et al., 2010; Cron et al., 2012).

Chloroplast genomes and rDNA have been widely utilized for constructing phylogenetic relationships and species identification in large, taxonomically challenging plant groups. Fu et al. (2024) demonstrated that both plastomes and nuclear rDNA (nrDNA) could significantly improve species resolution in *Acer*. Similarly, Chao et al. (2023) employed chloroplast genomes and 45S nrDNA to conduct phylogenetic analyses of *Bupleurum* in Sichuan Province, southwest China, providing strong evidence for the monophyly of each species.

In the family Marantaceae, Andersson (2001) inferred the phylogenetic relationships among 22 genera through nucleotide sequence variations of the *rps*16 intron and morphological characteristics. Prince and Kress (2006) conducted phylogenetic analyses using *mat*K, the 3’ intergenic spacer region, and the *trn*L-F intergenic spacer region. These analyses identified five major clades. Suksathan et al. (2009) explored the phylogenetic relationships of Asian Marantaceae using the *rps*16 intron, ITS1, and 5S-NTS, and determined that *Phrynium* is a non-monophyletic genus. However, the relationship between *Phrynium* and *Stachyphrynium* remains unclear, and most *Phrynium* species in China are lacking in these studies. Previous phylogenetic studies primarily focused on chloroplast DNA fragments and ITS regions rather than complete chloroplast genomes to address phylogenetic challenges within Marantaceae. In the context of *Phrynium* and *Stachyphrynium*, only the complete chloroplast genomes sequence of *P. rheedei* (a synonym of *P. pubinerve*) has been deposited in GenBank, without any accompanying publication or analysis. At present, there are several ongoing challenges, including an ambiguous understanding of the chloroplast genomes structure and characteristics of the Asian Marantaceae, a limited comprehension of interspecific relationships within *Phrynium* in China, and deficiency in effective molecular identification methods for both genera.

In this study, we newly sequenced the chloroplast genomes of seven *Phrynium* species and two *Stachyphrynium* species, covering nearly all representatives of these genera in China. Phylogenetic analysis of these taxa were conducted based on complete chloroplast genomes and ribosomal DNA (rDNA). This study aimed to: 1) characterize and compare structural variations in the chloroplast genomes of the newly sequenced species; 2) identify highly variable DNA barcodes for species delimitation within *Phrynium* and *Stachyphrynium*; 3) clarify the phylogenetic positions of *Phrynium* and *Stachyphrynium*, and resolve interspecific relationships within Chinese *Phrynium*.

Additionally, during a field investigation in Menghai County, Yunnan Province, China, a specimen unequivocally belonging to the genus *Phrynium* was collected. Its bracts are arranged in a pagoda-like formation, distinguishing it from other known Asian species. Through comprehensive field observations, literature review, specimen comparison and phylogenetic analysis, we finally identify it as a new species, formally described herein.

## 2 Materials and methods

### 2.1 Taxon sampling and morphological inspection

A total of 40 plant samples were collected, representing seven species of *Phrynium* (*P. pyramidale*, *P. pubinerve*, *P. imbricatum*, *P. hainanense*, *P. tonkinense*, *P. pedunculiferum* and *P. yunnanense*), two species of *Stachyphrynium* (*S. spicatum* and *S. placentarium*), and *Marantochloa purpurea*. *Marantochloa purpurea* was selected as an outgroup from a closely related tribe (Prince and Kress, 2006; Suksathan et al., 2009). These samples were collected from diverse regions throughout China, specifically including Yunnan Province, Hainan Province, Guangxi Zhuang Autonomous Region, and Xizang Autonomous Region. The voucher specimens are deposited in the herbarium of Guangzhou University of Chinese Medicine (GUCM), with detailed information provided in **Table 1**.

**Table 1:** Voucher information and GenBank accession numbers of materials.

Taxon	Locality	Collection date	Voucher	GenBank accession number	
				cp genomes	nrDNA
*Phrynium pyramidale* Y. Tong & Z. Y. Lin	Menghai, Yunnan	2023/07/08	Tong Y 230708014	PQ835434	PQ869816
	Menghai, Yunnan	2020/08/21	Tong Y 20082111	PQ835428	PQ869815
	Mengla, Yunnan	2023/07/11	Tong Y 230711003	PQ835426	PQ869814
	Menglun, Yunnan	2023/04/01	Tong Y 23040101	PQ835430	PQ869817
	Malipo, Yunnan	2022/03/19	Guo XB 22031903	PQ835429	PQ869820
	Malipo, Yunnan	2023/07/15	Tong Y 230715021	PQ835433	PQ869818
	Hekou, Yunnan	2023/07/13	Tong Y 230713002	PQ835436	PQ869819
	Pingbian, Yunnan	2023/07/14	Tong Y 230714012	PQ835431	PQ869821
	Pingbian, Yunnan	2023/07/14	Tong Y 230714003	PQ835432	PQ869822
	Motuo, Xizang	2023/12/30	Liu BY 23123001	PQ835427	PQ869823
	Vietnam	2023/12/28	Liao S 20231277	PQ835435	PQ869824
*P. pubinerve* Blume	Mengla, Yunnan	2023/07/10	Tong Y 230710003	PQ835425	PQ865401
*P. tonkinense* Gagnep.	Malipo, Yunnan	2020/08/16	Tong Y 20081604	PQ835441	PQ865465
	Malipo, Yunnan	2023/02/24	Tong Y 23022410	PQ835437	PQ865467
	Malipo, Yunnan	2022/03/21	Guo XB 22032103	PQ835438	PQ869826
	Malipo, Yunnan	2022/03/19	Guo XB 22031904	PQ835439	PQ865470
	Honghe, Yunnan	2023/07/14	Tong Y 230714002	PQ835440	PQ865471
*P. imbricatum* Roxb.	Wanning, Hainan	2023/08/20	Lin ZY 230820003	PQ835419	PQ865473
	Wanning, Hainan	2023/08/20	Lin ZY 230820014	PQ835417	PQ865474
	Baoting, Hainan	2023/08/22	Lin ZY 230822004	PQ835416	PQ869827
	Baoting, Hainan	2023/08/21	Lin ZY 230821004	PQ835418	PQ869825
*P. hainanense* T. L. Wu & S. J. Chen	Wanning, Hainan	2023/08/20	Lin ZY 230820008	PQ835415	PQ865476
	Baoting, Hainan	2023/08/22	Lin ZY 230822005	PQ835414	PQ865477
*P. pedunculiferum* D. Fang	Napo, Guangxi	2023/10/29	Tong Y 231029010	PQ835423	PQ865478
	Napo, Guangxi	2020/07/04	Zeng SJ 4736	PQ835420	PQ865481
	Menghai, Yunnan	2020/08/19	Tong Y 20081911	PQ835424	PQ865480
	Menghai, Yunnan	2023/07/08	Tong Y 230708019	PQ835421	PQ865479
	Baoshan, Yunnan	2023/07/13	Tong Y 230713004	PQ835422	PQ865483
*P. yunnanense* Y. S. Ye & L. Fu	Hekou, Yunnan	2020/08/17	Tong Y 20081708	PQ835442	PQ865482
*Stachyphrynium* *spicatum* (Roxb.) K. Schum.	Mengla, Yunnan	2024/09/27	Lin ZY 24092701	PQ835452	PQ865484
*S. placentarium* (Lour.) Clausager & Borchs.	Baoting, Hainan	2023/08/22	Lin ZY 230822002	PQ835445	PQ865485
	Wanning, Hainan	2023/08/20	Lin ZY 230820011	PQ835450	PQ869828
	Malipo, Yunnan	2020/08/16	Tong Y 20081603	PQ835443	PQ865486
	Hekou, Yunnan	2023/08/18	Tong Y 20081808	PQ835444	PQ865488
	Mengla, Yunnan	2023/07/09	Tong Y 230709016	PQ835449	PQ865489
	Xichou, Yunnan	2023/02/22	Tong Y 23022228	PQ835447	PQ865490
	Longzhou, Guangxi	2023/04/25	Tong Y 23042503	PQ835448	PQ865491
	Napo, Guangxi	2020/08/14	Tong Y 20081409	PQ835446	PQ865492
	Zhaoqing, Guangdong	2019/06/02	Tong Y 19060201	PQ835451	PQ869829
*Marantochloa purpurea* (Ridl.) Milne-Redh.	Guangzhou (cultivated), Guangdong	2023/11/12	Tong Y 23111201	PQ835413	PQ865494

Key morphological characteristics of taxonomic significance in *Phrynium* were documented through photography, measurements, and the recording of variation ranges. This analysis focused on sheath, leaf shape and size, bract shape, texture and arrangement, as well as flower size and color, and the shape and size of sepals, with particular emphasis on comparing *P. pyramidale* to the two related species.

### 2.2 DNA extraction and genome sequencing

Genomic DNA was extracted from 50 mg of silica gel-dried leaves using a modified CTAB method (Doyle and Doyle, 1987). The purity and integrity of the isolated DNA were evaluated using the B-500 ultra-micro spectrophotometer (Metash, China) and agarose gel electrophoresis. High-quality genomic DNA was randomly fragmented into 300-400 bp segments using ultrasound treatment for library construction. Sequencing was performed on the DNBSEQ platform (150 bp paired-end) at BGI-Wuhan, generating a total of 3.0 Gb of clean data for each sample.

### 2.3 Assembly and annotation

The chloroplast genomes assembly was conducted using GetOrganelle v1.7.5.0 (Jin et al., 2020), with *Phrynium rheedei* (MZ958828.1) and *Thalia dealbata* (PP059847.1) as reference genomes. The assembly of nrDNA followed the same methodology.

Preliminary annotation was executed using Plastid Genome Annotator (PGA) software (Qu et al., 2019). The results of the annotation were subsequently analyzed and refined through manual corrections in Geneious Prime R9.0.2 (Kearse et al., 2012), which included the identification of start and stop codons for each expressed gene, in comparison to the reference genome. Transfer RNAs (tRNAs) were identified using tRNAscan-SE v2.0.7 (Chan et al., 2021). The comprehensive chloroplast maps were generated with OGDRAW (Lohse et al., 2007), and the complete chloroplast sequences were submitted to GenBank (http://www.ncbi.nlm.nih.gov/). The annotation of nrDNA was also conducted using the same software, with comparisons made to the reference genome.

### 2.4 Genome structure analysis

Geneious R9.0.2 was used to determine the total length of the chloroplast genomes sequences of all species within the genera *Phrynium* and *Stachyphrynium*, as well as to assess the lengths of LSC, IR, SSC regions, along with GC content in each of these regions. The annotated chloroplast genomes sequences were subsequently uploaded to the IRscope online software (Amiryousefi et al., 2018) for a visual analysis of the contraction and expansion of the IR boundaries, resulting in the generation of simplified diagrams.

### 2.5 Codon usage analysis

PhyloSuite v1.2.2 (Zhang et al., 2020) was employed to extract protein-coding sequences (CDS) from chloroplast genomes, with sequences less than 300 bp being excluded to enhance the accuracy of the results. The relative synonymous codon usage (RSCU) of these sequences was analyzed using CodonW v.1.4.2 (http://codonw.sourceforge.net/), and the values of RSCU were utilized to generate the heat maps using TBtools-II v2.119 (Chen et al., 2023). A codon exhibiting an RSCU value exceeding 1 signifies a higher frequency of usage, while a value below 1 indicates less frequent usage (Sharp and Li, 1987).

### 2.6 Simple sequence repeat analysis

MISA-web (http://pgrc.ipk-gatersleben.de/misa) was employed to identify the simple sequence repeats (SSRs) within chloroplast genomes. The parameters for the minimum repeated size were established for mononucleotides to hexanucleotides at thresholds of 10, 5, 4, 3, 3 and 3, respectively (Beier et al., 2017).

### 2.7 Comparative analysis

The Mauve v.2.4.0 plugin (Darling et al., 2004) in Genious R9.0.2. was used to conduct multiple sequence alignments for the collinearity analysis of *Phrynium* and *Stachyphrynium*, with the aim of identifying potential rearrangements and inversions. Comparative analysis of the chloroplast genomes was carried out using mVISTA (Frazer et al., 2004) online program (https://genome.lbl.gov/vista/index.shtml), utilizing the Shuffle-LAGAN mode to illustrate the variations present among the genomes. Additionally, the sliding window analysis of the whole chloroplast genomes and nrDNA were executed using DnaSP v6.0 (Rozas et al., 2017) to determined the nucleotide diversity (Pi). For chloroplast genomes, 600 bp window length and 200 bp step size were employed, while for nrDNA, 50 bp window length and 20 bp step size was utilized.

### 2.8 Phylogenetic analysis

Phylogenetic relationships were reconstructed using Bayesian inference (BI) and maximum likelihood (ML) methods based on CDS and nrDNA sequences. In addition to the sequences from *Phrynium* and *Stachyphrynium*, the outgroup *Marantochloa purpurea* of Marantaceae, cultivated in China, was incorporated into the phylogenetic analysis. (**Table 1**). Sequences were aligned using MAFFT v7 (Katoh and Standley, 2013) with default parameters and then trimmed with trimAL (Capella-Gutierrez et al., 2009) in TBtools, which served to remove poorly aligned regions and enhance the accuracy of the resulting.

For the ML analyses, the optimal nucleotide substitution model was determined using ModelFinder (Kalyaanamoorthy et al., 2017) based on the Akaike Information Criterion (AIC). ML trees were constructed using IQ-TREE v1.6.1 (Lanfear et al., 2020) with 1,000 bootstrap replicates. For the BI analyses, jModelTest2 (Darriba et al., 2012) was used for the model selection with Bayesian Information Criterion (BIC) as the standard, and MrBayes v3.2.7 (Ronquist et al., 2012) was utilized to construct the BI trees with default settings. The Markov Chain Monte Carlo (MCMC) analysis was run for 2 million generations, with tree sampling occurring every 1,000 generations. Convergence of the MCMC was assumed when the average standard deviation of split frequencies (ASDF) fell below 0.01. The initial 25% of the trees were discarded due to the unreliability of early MCMC sampling data, and the remaining trees were used to generate consensus trees and estimate Bayesian posterior probabilities (PPs).

## 3 Results

### 3.1 Features of chloroplast genome and nrDNA

**Figure 1:**
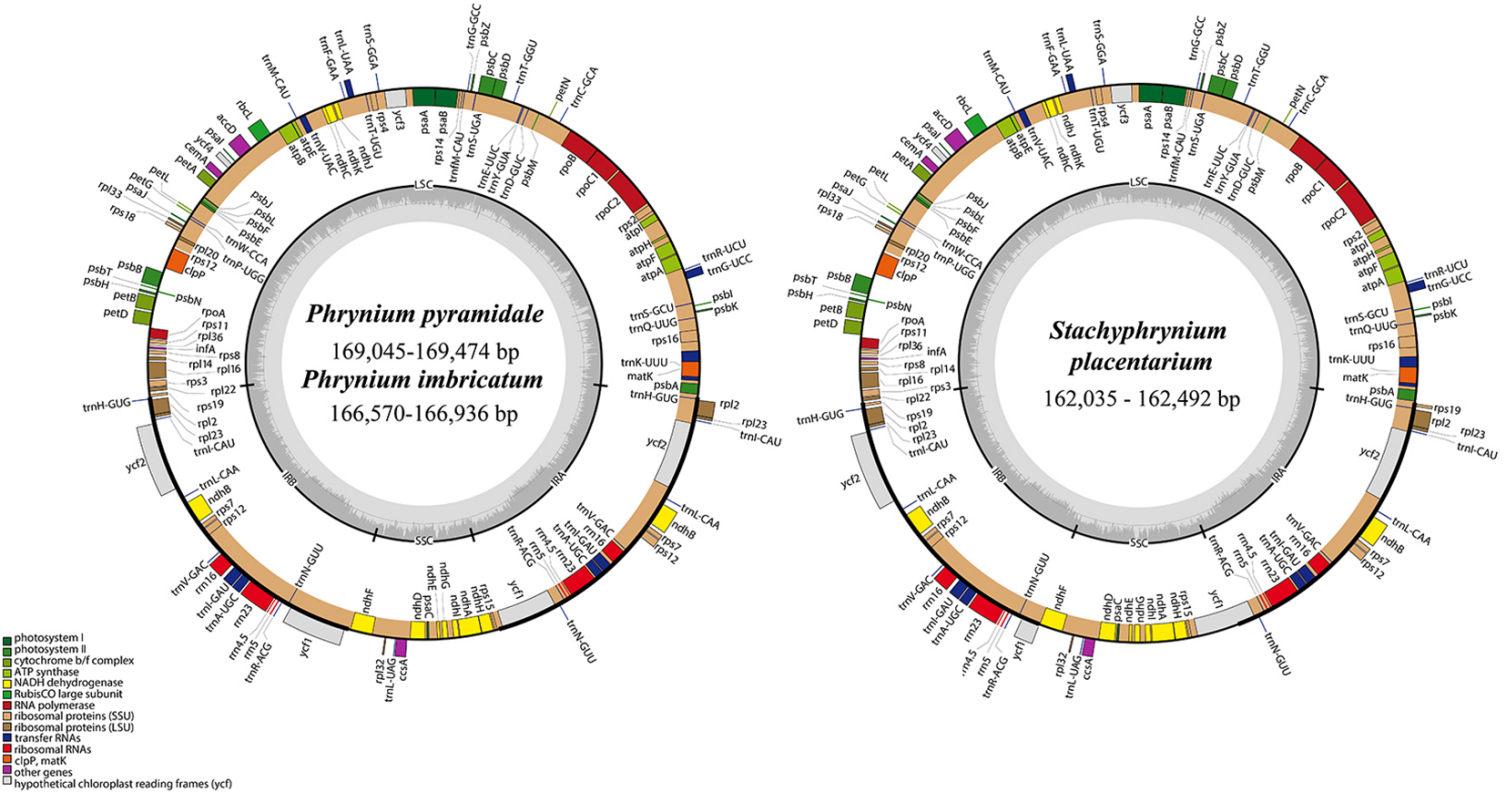
The complete chloroplast genome maps of two *Phrynium* and one *Stachyphrynium* species. The inner circle represents the quadripartite structure, with a large single copy (LSC), a small single copy (SSC) and two inverted repeat regions (IRA and IRB) which the GC content in dark gray, and the AT content in light gray. Genes shown inside the circle are transcribed clockwise, and those outside the circle are transcribed anti-clockwise.

In this study, 40 chloroplast genomes were newly sequenced and assembled, including 30 from *Phrynium* and 10 from *Stachyphrynium*. The genome size of *Phrynium* ranged from 166,401 bp (*P. tonkinense*) to 172,065 bp (*P. pedunculiferum*) (**Table 2**). The chloroplast genome maps for two species of *Phrynium* and one species of *Stachyphrynium* are shown in **Figure 1**, with the remaining species are provided in the supplementary materials (**Supplementary Figure 1**). These genomes exhibit a characteristic quadripartite structure, consisting of a pair of IR regions (26,258-35,228 bp) that are separated by the LSC (89,787-92,551 bp) and SSC (11,071-21,631 bp) regions, with the SSC region displaying the most significant variation in length. The total GC content varied from 35.8 to 36.4%, with a predominant concentration at 36.2%. Generally, the GC content in the IR regions (38.8-42.8%) is higher than that in both the LSC region (33.9-34.3%) and the SSC region (28.6-30.9%). In contrast, the genome lengths of *Stachyphrynium* are notably shorter than those of *Phrynium*, ranging from 162,035 bp (*S. placentarium*) to 167,870 bp (*S. spicatum*). The observed variation in sequence length can be primarily attributed to differences in the lengths of the SSC region (14,291-18,877 bp) and the IR regions (27,471-32,069 bp). The overall GC content exhibited a high degree of similarity (36.2-36.5%), with specific GC content values of 40.1% to 42.2% in the IR regions, 34.2% to 34.4% in the LSC region, and 29.9% to 30.8% in the SSC region (**Table 2**).

**Table 2:** Summary of the complete chloroplast genomes characteristics of *Phrynium* and *Stachyphrynium* species.

Species	Total length (bp) GC (%)	LSC length (bp) GC (%)	SSC length (bp) GC (%)	IR length (bp) GC (%)	Number of genes		
					Protein	tRNA	rRNA
*P. pyramidale*	169,045-169,474 36.2	91,223-91,494 34.1	14,868-14,928 30.0-30.1	31,362-31,660 40.5-40.6	86	30	4
*P. pubinerve*	171,807 35.8	90,281 34.2	11,071 29.6	35,228 38.8	86	30	4
*P. tonkinense*	166,401-166,815 36.0-36.1	91,878-92,551 33.9	20,656-20,859 28.8-29.0	26,258-27,037 42.4-42.8	87	30	4
*P. imbricatum*	166,570-166,936 36.4	89,787-90,003 34.3	14,129-14,147 30.9	31,219-31,501 40.5-40.7	86	30	4
*P. hainanense*	166,773-166,843 36.1	91,838-91,883 34	20,806-20,830 28.8-28.9	27,065 42.4	87	30	4
*P. pedunculiferum*	171,625-172,065 35.9	91,339-91,864 34.0	15,536-15,614 29.1-29.3	32,304-32,375 40.1	87	30	4
*P. yunnanense*	166,694 36.0	91,858 33.9	21,631 28.6	26,602 42.5	87	30	4
*S. spicatum*	167,870 36.2	89,441 34.2	14,291 30.8	32,069 40.1	87	30	4
*S. placentarium*	162,035-162,492 36.5	88,228-88,566 34.2-34.4	18,725-18,877 29.9	27,471-27,606 42.2	87	30	4

The chloroplast genomes of *Phrynium* and *Stachyphrynium* demonstrate a significant degree of similarity, with gene arrangements maintained in the same order. These genomes encoded 112-113 unique genes, which include 86-87 protein-coding genes (PCGs), 30 tRNA genes, and 4 ribosomal RNA genes (**Table 3**). A gene count revealed that *P. imbricatum* and *P. pyramidale* contained a single copy of the *rps*19 within the LSC region, whereas other species exhibited two copies in each IR region. Additionally, a pseudogene *ycf*1 was identified in *P. hainanense*, *P. tonkinense*, *P yunnanense* and *S*. *placentarium*, while the *ndh*A gene was found to be deleted in *P. pubinerve* (**Figure 1**; **Supplementary Figure 1**).

**Table 3:** Gene contents of *Phrynium* and *Stachyphrynium* complete chloroplast genomes.

Gene function	Gene category	Name of gene
Self-replication	Large subunit of ribosome	*rpl*2^a,*^×2, *rpl*14, *rpl*16^*^, *rpl*20, *rpl*22, *rpl*23^a^×2, *rpl*32, *rpl*33, *rpl*36
	Small subunit of ribosome	*rps*2, *rps*3, *rps*4, *rps*7^a^×2, *rps*8, *rps*11, *rps*12 ^a,b,**^×2, *rps*14, *rps*15, *rps*16^*^, *rps*18, *rps*19^a^×2
	DNA-dependent RNA polymerase	*rpo*A, *rpo*B, *rpo*C1^*^, *rpo*C2
	Ribosomal RNAs	*rrn*4.5^a^×2, *rrn*5^a^×2, *rrn*16^a^×2, *rrn*23^a^×2
	Transfer RNAs	*trn*A-UGC^a,*^×2, *trn*C-GCA, *trn*D-GUC, *trn*E-UUC, *trn*F-GAA, *trn*fM-CAU, *trn*G-GCC, *trn*G-UCC^*^, *trn*H-GUG^a^×2, *trn*I-CAU ^a^×2, *trn*I-GAU^a,*^×2, *trn*K-UUU^*^, *trn*L-CAA^a^×2, *trn*L-UAA^*^, *trn*L-UAG, *trn*M-CAU, *trn*N-GUU^a^×2, *trn*P-UGG, *trn*Q-UUG, *trn*R-ACG^a^×2, *trn*R-UCU, *trn*S-GCU, *trn*S-GGA, *trn*S-UGA, *trn*T-GGU, *trn*T-UGU, *trn*V-GAC^a^×2, *trn*V-UAC^*^, *trn*W-CCA, *trn*Y-GUA
Photosynthesis	Photosystem I	*psa*A, *psa*B, *psa*C, *psa*I, *psa*J
	Photosystem II	*psb*A, *psb*B, *psb*C, *psb*D, *psb*E, *psb*F, *psb*H, *psb*I, *psb*J, *psb*K, *psb*L, *psb*M, *psb*N, *psb*T, *psb*Z, *ycf*3^**^
	Subunits of NADH-dehydrogenase	*ndh*A^*^, *ndh*B^a,*^×2, *ndh*C, *ndh*D, *ndh*E, *ndh*F *ndh*G, *ndh*H, *ndh*I, *ndh*J, *ndh*K
	Subunits of cytochrome b/f complex	*pet*A, *pet*B^*^, *pet*D^*^, *pet*G, *pet*L, *pet*N
	ATP synthase	*atp*A, *atp*B, *atp*E, *atp*F^*^, *atp*H, *atp*I
	Large subunit of rubisco	*rbc*L
Other genes	Translation initiation factor	*inf*A
	Maturase	*mat*K
	Protease	*clp*P^**^
	Envelope membrane protein	*cem*A
	Subunit of accetyl-CoA-carboxylase	*acc*D
	Cytochromesynthesis	*ccs*A
Genes of unknown function		*ycf*1^a^×2, *ycf*2^a^×2, *ycf*4

**^a^** Genes in inverted repeat regions; **^b^** trans-splinting gene; ^*^ genes containing one intron; ^**^ genes containing two introns; **×2** shows genes with duplicates.

The nrDNA sequence lengths of seven species of *Phrynium* are 5,792-5,821 bp comprising five fragments, i.e., the 18S rRNA gene, ITS1, 5.8S rRNA gene, ITS2 and the 26S rRNA gene. The lengths of these fragments are 1,811 bp, 195-206 bp, 164 bp, 207-229 bp, and 3,411-3,414 bp, respectively. The overall nrDNA GC content is 57.2-57.6%, with the GC content in the ITS regions being the highest among the five fragments (65.1-68.0%). The total length of the nrDNA in *Stachyphrynium*, along with the lengths of its individual components, closely resembles that of the *Phrynium* (**Table 4**).

**Table 4:** Summary of nrDNA (18S-ITS1-5.8S-ITS2-26S) characteristics of *Phrynium* and *Stachyphrynium* species.

Species	Total length (bp)	18S rRNA length (bp)	ITS1 length (bp)	5.8S rRNA length (bp)	ITS2 length (bp)	26S rRNA length (bp)	Total GC content (%)	ITS GC content (%)
*P. pyramidale*	5,812-5,813	1,811	202	164	222-223	3,413	57.4-57.5	66.9-67.1
*P. pubinerve*	5,821	1,811	206	164	229	3,411	57.6	67.4
*P. tonkinense*	5,792-5,797	1,811	195-198	164	207-211	3,413	57.4	65.7-66.2
*P. imbricatum*	5,800	1,811	202	164	210	3,413	57.2-57.3	65.1
*P. hainanense*	5,795-5,798	1,811	195	164	212-215	3,413	57.5	66.9-67.2
*P. pedunculiferum*	5,805-5,808	1,811	195-198	164	220-221	3,414	57.4-57.6	66.0-67.6
*P. yunnanense*	5,799	1,811	195	164	216	3,413	57.6	68.0
*S. spicatum*	5,809	1,810	195	164	231	3,409	57.6	68.6
*S. placentarium*	5,806	1,810	195	164	230	3,407	57.6	66.5-67.2

### 3.2 Contraction and expansion of IR regions

**Figure 2:**
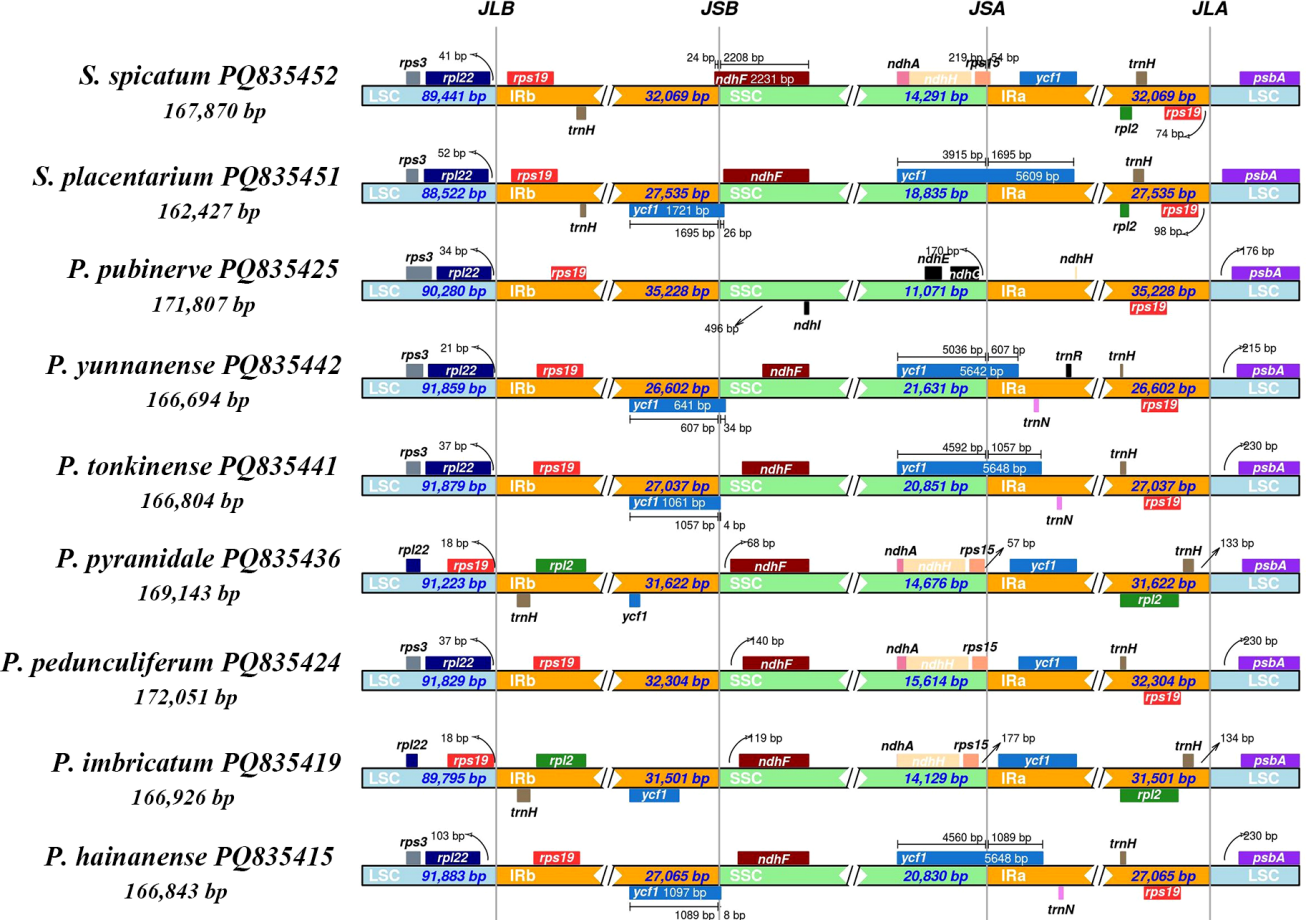
Comparison of the LSC, SSC and IR junction among the seven *Phrynium* and two *Stachyphrynium* species. JLB, junction of LSC/IRb; JSB, junction of IRb/SSC; JSA, junction of SSC/IRa; JLA, junction of IRa/LSC.

To investigate the mechanism driving variation in chloroplast genomes, we conducted a detailed comparative analysis of the four junctions (JLB, JSB, JSA, and JLA) between the LSC, SSC, and two IR regions across seven species of *Phrynium* and two species of *Stachyphrynium*. This analysis focused on the locations of the IR boundaries and their adjacent genes. The findings indicate that the two junctions between the LSC and IR regions, JLB (LSC/IRb) and JLA (IRa/LSC) exhibit relative stability. In contrast, the junctions JSB (IRb/SSC) and JSA (SSC/IRa) display greater variability among the species examined (**Figure 2**). With the exception of the JLBs found in *P. pyramidale* and *P. imbricatum*, which were are situated between *rps*19 and *trn*H genes, all other JLBs in *Phrynium* and *Stachyphrynium* species were located between the *rpl*22 and *rps*19 genes. The *rpl*22 gene was identified in the LSC region, positioned 21-103 bp from the IRb region, while the *rps*19 gene was found 18 bp from the JLB instead of *rpl*22. Similarly, aside from the JLAs of *P. pyramidale* and *P. imbricatum*, which were located between the *trn*H and *psb*A genes, all other types were positioned between the *rps*19 and *psb*A genes. In *Stachyphrynium*, JLAs were found 74 to 98 bp from *rps*19, whereas in *Phrynium*, they were loacted 176 to 230 bp from *psb*A. In the chloroplast genomes of *P. hainanense*, *P. tonkinense*, *P. yunnanense* and *S.placentarium*, the *ycf*1 gene expanded from the SSC to the IRa region, with a range of 607-1,695 bp. Additionally, the *ycf*1 gene was also present in the IRb region in these species due to partial duplication. This truncation likely results in a nonfunctional protein, leading to the classification of *ycf*1 as a pseudogene. In the remaining species, *ycf*1 was entirely located within the IRa region and being duplicated. Regarding the *rps*19 gene, it was entirely situated within the IR regions in all species, with the exception of *P. pyramidale* and *P. imbricatum*, where it was located in the LSC region.

### 3.3 Relative synonymous codons usage

**Figure 3:**
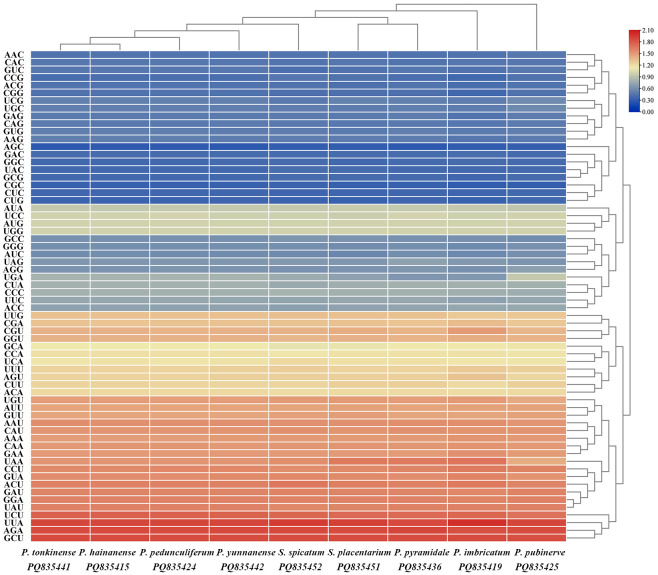
Heatmap of relative synonymous codon usage (RSCU) values for seven *Phrynium* and two *Stachyphrynium* species.

The analysis of codon usage frequency and relative synonymous codon usage (RSCU) was conducted using protein-coding genes. The protein-coding genes in the chloroplast genomes of the seven *Phrynium* species comprised between 18,329-20,711 codons, encoding a total of 20 amino acids, while the two *Stachyphrynium* species contained between 20,332-20,432 codons. The results indicate that RSCU values were comparable across different genera (**Figure 3**). The codons UAA, UGA and UAG were identified as stop codons. The RSCU analysis revealed that nearly all amino acids, with the exception of methionine (Met) and tryptophan (Trp), were represented by 2 to 6 synonymous codons. Among the 64 codons analyzed for the *Phrynium* and *Stachyphrynium* species, 30 exhibited RSCU values exceeding 1, indicating a preference for certain codons, with the majority (29/30, 96.67%) end with the base A or U. Similarly, 31 codons had RSCU values below 1, with most (28/31, 90.32%) end with the base C or G. The codons AUG and UGG, which correspond to methionine and tryptophan respectively, displayed no significant bias (RSCU = 1.00). Notably, the UUA condon, which encodes leucine, exhibited the highest RSCU value of approximately 1.90, while the AGC codon, encoding serine, demonstrated the lowest RSCU value at approximately 0.29.

### 3.4 Simple sequence repeat analysis

**Figure 4:**
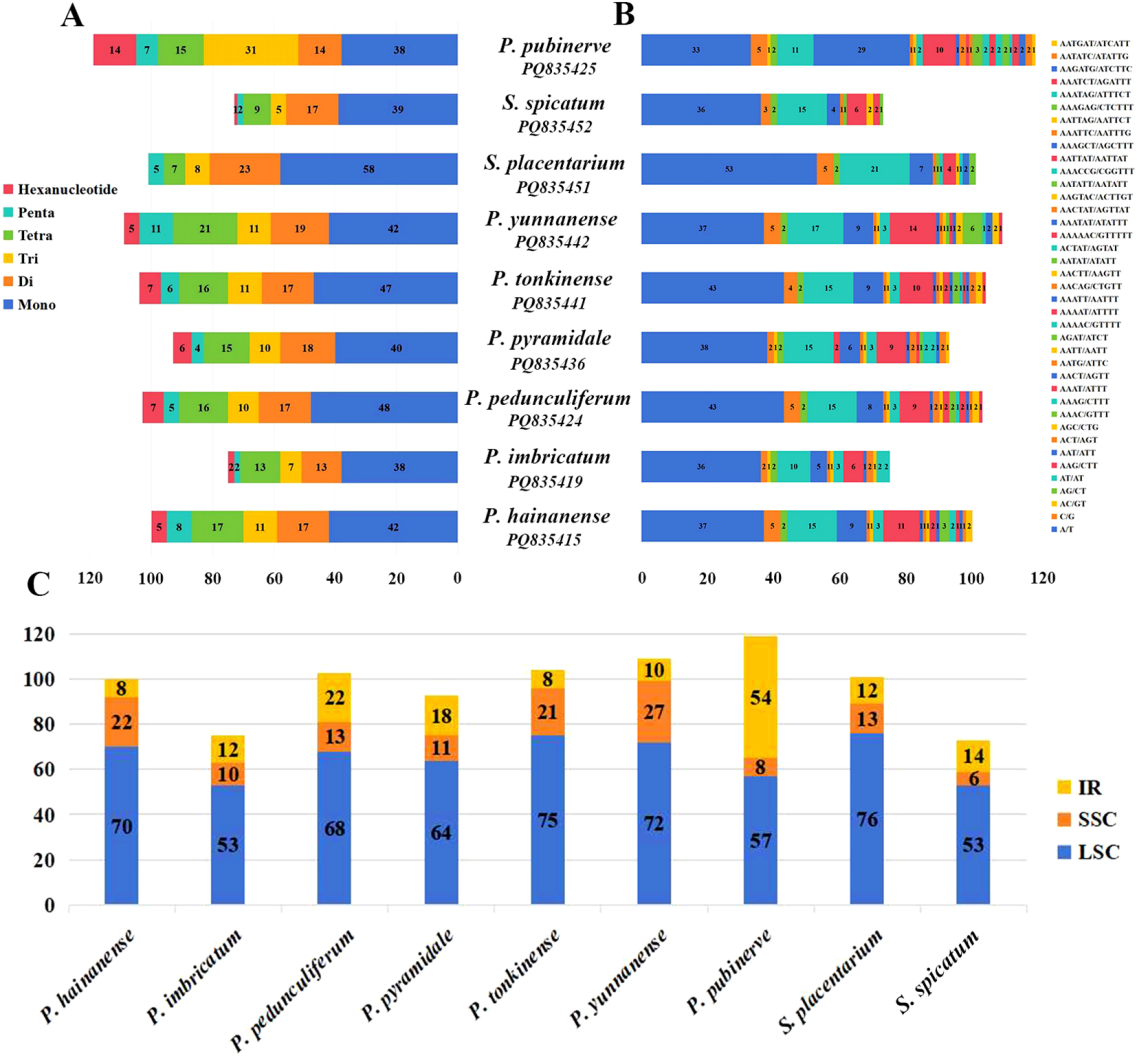
Analysis of SSRs in the chloroplast genomes of *Phrynium* and *Stachyphrynium*. **(A)** The number of six different SSR types; **(B)** The number of different SSR repeat units; **(C)** The number of SSRs distributed in LSC, SSC and IR regions.

A total of 75 to 119 SSRs were identified in chloroplast genomes of *Phrynium* (**Figure 4**). The average percentages of SSRs, ranging from mononucleotides to hexanucleotides, were 41.96%, 16.36%, 12.95%, 16.07%, 6.12%, and 6.54%, respectively. Notably, SSRs composed of A/T bases represented a significant proportion (27.73-48.0%). The distribution of SSRs in chloroplast was observed to be uneven, with 65.3% of SSRs located in the LSC region, 15.93% in the SSC region, and 18.78% in the IR regions. This distribution indicates a higher level of polymorphic variation within the LSC region (**Figure 4**). However, the proportion of SSRs in the IR region increased (45.38%) in *P. pubinerve*. In the case of *Stachyphrynium*, a total of 73-101 SSRs were identified, with no hexanucleotide repeats detected in *S. placentarium*. The predominant type of repeat observed was mononucleotides, which constituted approximately 53.42-57.43% of the total SSR loci. Consistent with the findings in *Phrynium*, the majority of SSRs in *Stachyphrynium* were also distributed in the LSC region (74.14%).

### 3.5 Comparative analysis

**Figure 5:**
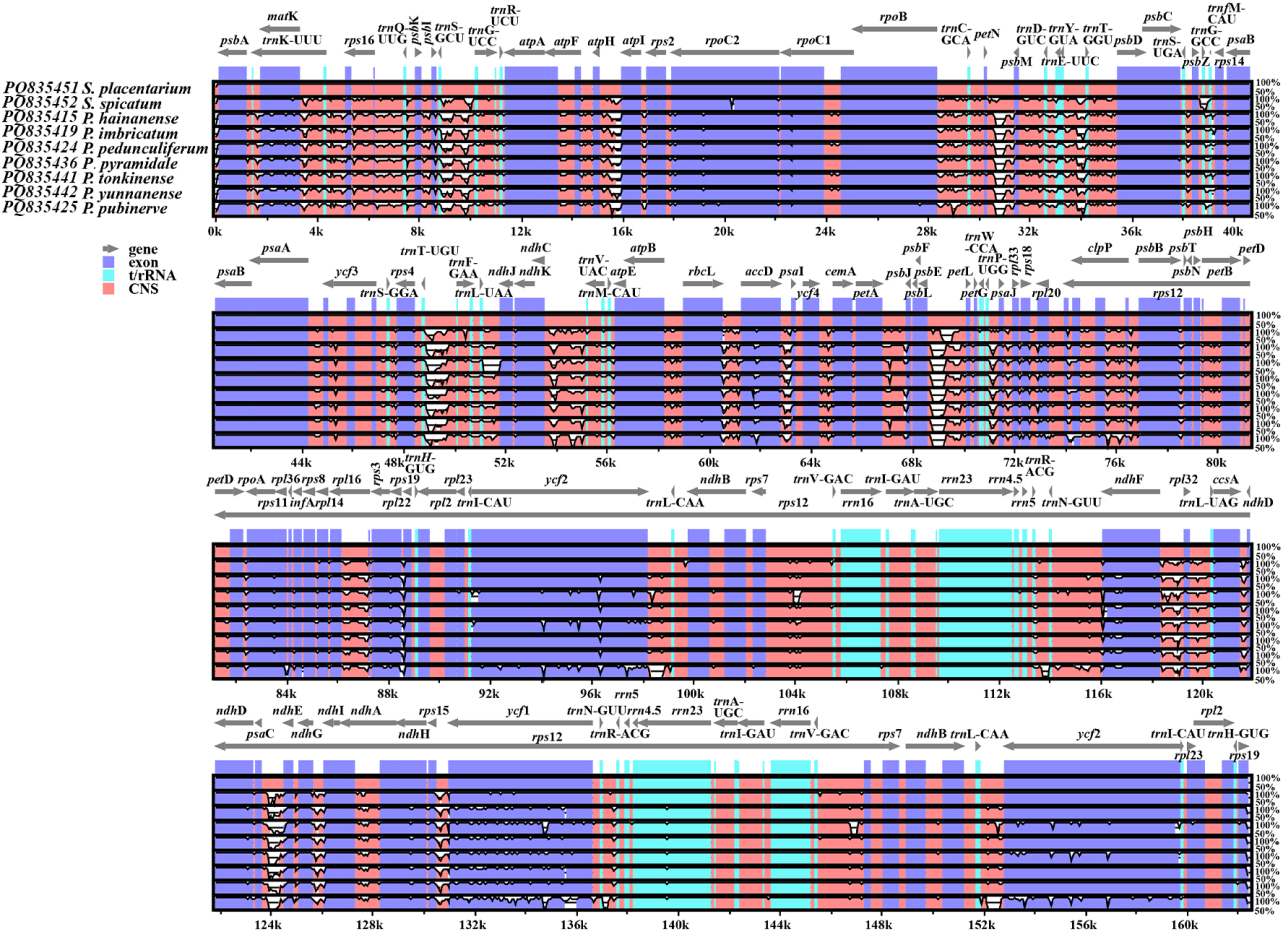
Comparison of seven *Phrynium* and two *Stachyphrynium* species of chloroplast genomes with *S. placentarium* as a reference sequence. Gray arrows above the alignment indicate the orientation of each gene. The colored areas indicate the exon, t/rRNA, and conserved non-coding sequences (CNS). The vertical axis indicates the percentage of identity, ranging from 50 to 100%.

The multiple sequence alignments performed with mVISTA software revealed a notable similarity in the complete chloroplast genomes of the examined species of *Phrynium* and *Stachyphrynium*. The results (**Figure 5**) indicated that the non-coding regions of the chloroplast genomes exhibited a higher frequency of variation compared to the coding regions, suggesting that sequence diversity is more pronounced in the non-coding regions. The SSC and LSC regions exhibited significantly greater variability compared to the IR regions, while the rRNA genes remained highly conserved, showing minimal variation. Among the coding regions, the *ycf*1 and *ycf*2 gene were identified as highly variable, whereas the non-coding regions, including *trn*S-*trn*G, *atp*H-*atp*I, *pet*N-*psb*M, *trn*E-*trn*T, *rps*4-*trn*L, *psb*E-*pet*L, *acc*D-*psa*I, *ndh*F-*rpl*32, *psa*C-*ndh*I and *ndh*H-*ycf*1, exhibited a higher degree of differentiation.

**Figure 6:**
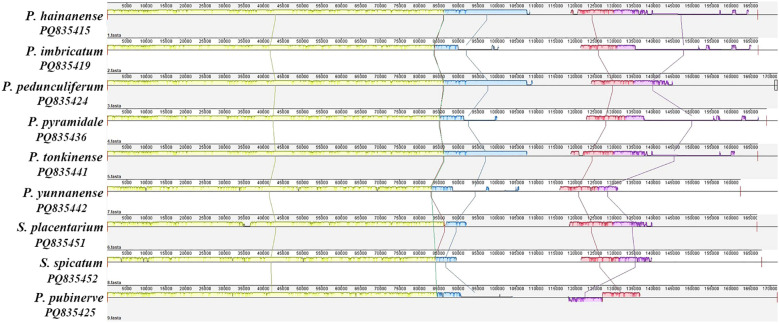
Mauve alignment of the whole chloroplast genomes in seven *Phrynium* and two *Stachyphrynium* species.

The collinearity analysis of *Phrynium* and *Stachyphrynium*, conducted using Mauve, demonstrated the presence of locally collinear blocks (LCBs) within these chloroplast genomes (**Figure 6**). The LSC region and its adjacent regions exhibited a greater consistency in gene order compared to the SSC and IR regions. A significant distinction was observed in *P. pubinerve*, characterized by an inversion of an approximately 8 kb segment that spans from *ndh*F to *ndh*I, transitioning from the SSC to the IR region, with the inversion occurring at the junction of IRb/SSC.

### 3.6 Sequence divergence and mutational hotspots

**Figure 7:**
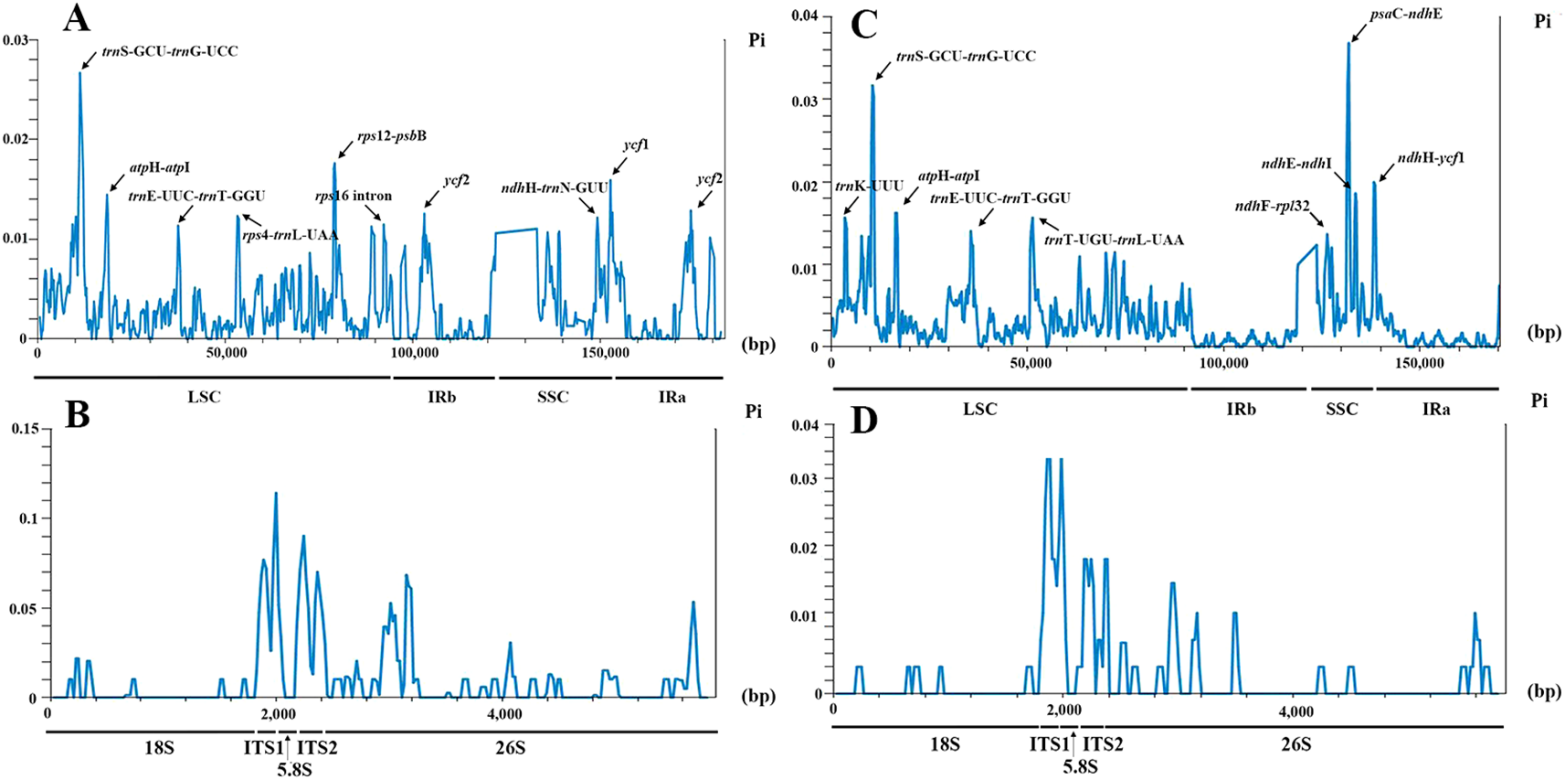
Sliding window analysis of seven *Phrynium* and two *Stachyphrynium* chloroplast genomes and nrDNA alignment. X-axis: position of the midpoint of a window. Y-axis: nucleotide diversity (Pi) of each window. **(A)** *Phrynium* chloroplast genomes; **(B)** *Phrynium* nrDNA; **(C)** *Stachyphrynium* chloroplast genomes; **(D)** *Stachyphrynium* nrDNA.

Nucleotide diversity (Pi) values of the complete chloroplast genomes and nrDNA were assessed to identify hot spots of sequence divergence in *Phrynium* and *Stachyphrynium*. The sliding window analysis revealed that the Pi values of the chloroplast genomes of seven *Phrynium* species varied from 0-0.02669 over a 600 bp region, with greater divergence observed in LSC and SSC regions compared to the IR regions. A total of ten highly variable loci were identified in the chloroplast genomes of *Phrynium*, including *trn*S-GCU-*trn*G-UCC (0.02669), *rps*12-*psb*B (0.01761), *ycf*1 (0.01592), *clp*P (0.01592), *atp*H-*atp*I (0.01444), *ycf*2 (0.01287), *rps*4-*trn*L-UAA (0.01232), *ndh*H-*trn*N-GUU (0.01216), *rpl*16 intron (0.01147), and *trn*E-UUC-*trn*T-GGU (0.01135). The intergenic spacer regions exhibited significantly higher Pi values compared to the coding regions, with only two coding regions (*ycf*1 and *clp*P) displaying elevated values (**Figure 7A**). The nrDNA sequences from the seven *Phrynium* species contained 170 variable sites and 146 parsimony-informative sites, accounting for 2.91% and 2.50% of the total sequences, respectively. The figure (**Figure 7B**) shows that the majority of the variation is concentrated in the ITS1 region. The Pi values of nrDNA ranged from 0-0.11429, with average values of 0.05982 for ITS1, 0.05321 for ITS2, 0.0104 for 5.8S rRNA, 0.00201 for 18S rRNA and 0.00779 for 26S rRNA.

A similar pattern was observed in *Stachyphrynium*, where regions of the chloroplast genomes with elevated Pi values included *psa*C-*ndh*E (0.03678), *trn*S-GCU-*trn*G-UCC (0.03167), *ndh*H-*ycf*1 (0.01993), *ndh*E-*ndh*I (0.01856), *atp*H-*atp*I (0.01622), *trn*K-UUU (0.01563), *trn*T-UGU-*trn*L-UAA (0.01563), *trn*E-UUC-*trn*T-GGU (0.014), and *ndh*F-*rpl*32 (0.01363) (**Figure 7C**). The nrDNA sequences from the two *Stachyphrynium* species contained 69 variable sites, representing 1.18% of the total sequence. The Pi values of nrDNA ranged from 0-0.03678, with the average values of 0.02502 for ITS1, 0.01381 for ITS2, 0.0025 for 5.8S rRNA, 0.00067 for 18S rRNA and 0.00145 for 26S rRNA (**Figure 7D**).

### 3.7 Phylogenetic analysis

**Figure 8:**
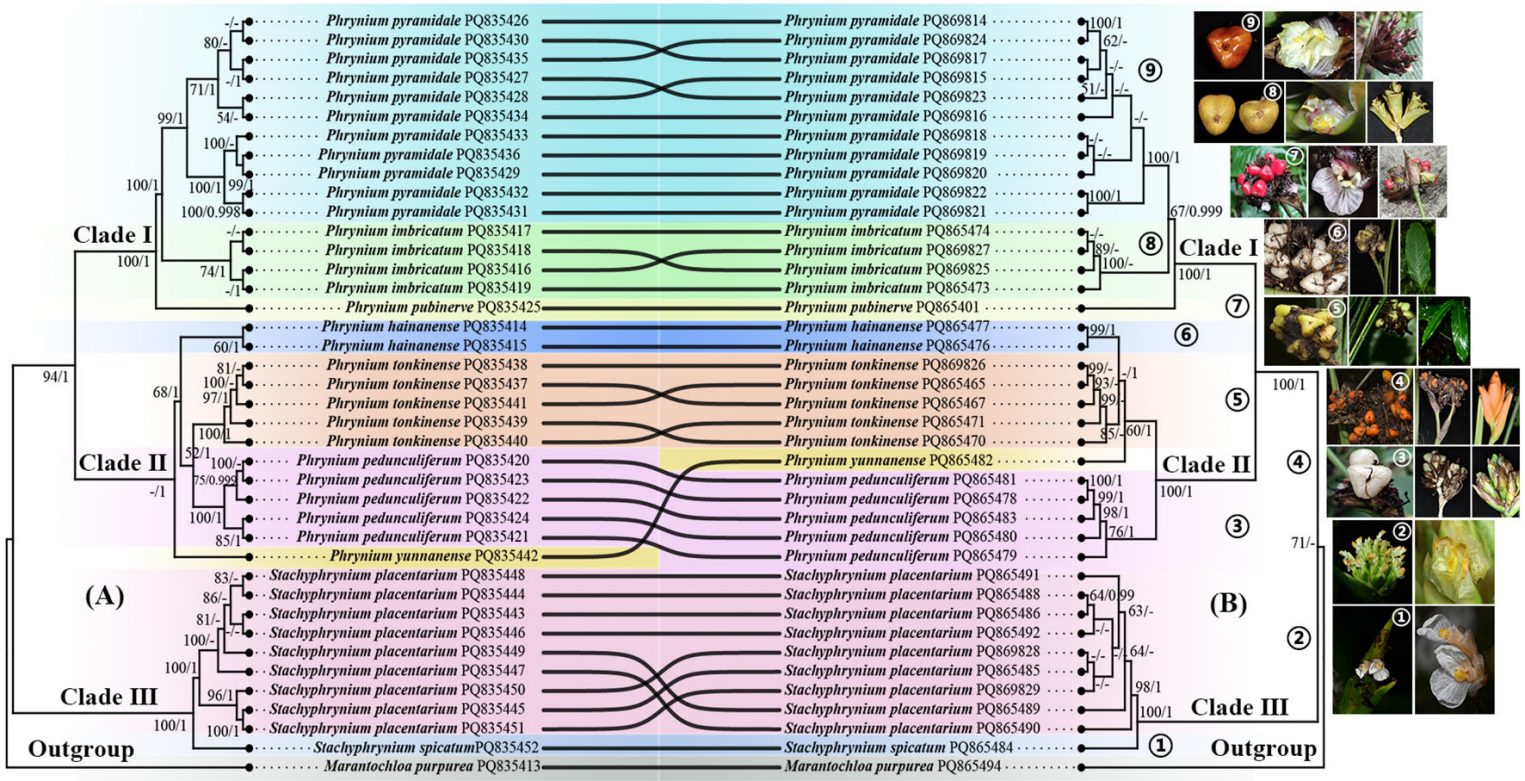
Comparison between phylogenetic trees based on CDS **(A)** and nrDNA sequences **(B)**. Numbers on each branch represent BS (bootstrap support)/PP (posterior probability); “-” represents incongruences of BI and ML trees.

Phylogenetic trees were constructed using the Maximum Likelihood (ML) and Bayesian Inference (BI) methodologies, based on the analysis of CDS and nrDNA sequences, with *Marantochloa purpurea* (Marantaceae) serving as the outgroup (**Figure 8**). The topology of the ML and BI trees demonstrated a high degree of similarity, and several nodes on these trees received strong support. The Chinese *Phrynium* species were grouped into a monophyletic clade, which was sister to that of *Stachyphrynium*. This clade could be further subdivided into two distinct clades: one consisting of *P. pyramidale*, *P. pubinerve* and *P. imbricatum* (BS=100%, PP=1.00), and another encompassing all remaining Chinese species. The position of *P. yunnanense* varied across different trees. In the CDS tree, it formed an independent branch that was sister to other species, situated adjacent to *P. pedunculiferum* and *P. tonkinense*. Conversely, in the nrDNA tree, *P. yunnanense* exhibited a closer relationship with *P. pedunculiferum*, subsequently merging with *P. hainanense* and *P. tonkinense*. Additionally, the *Stachyphrynium* species in China also constituted a monophyletic clade.

## 4 Discussion

### 4.1 General characteristics of the chloroplast of *Phrynium* and *Stachyphrynium*

The chloroplast genomes of seven *Phrynium* and two *Stachyphrynium* species, newly reported in this study, exhibits a significant degree of similarity regarding genome structure, gene order, and gene composition. The distribution of GC content in the chloroplast genomes was uneven, with elevated GC content observed in the IR regions compared to the LSC and SSC regions. This difference may be explained by the presence of four types of rRNAs (*rrn*4.5, *rrn*5, *rrn*16, and *rrn*23) in the IR region, while the reduced GC content in the SSC region is likely due to the presence of NADH dehydrogenase genes, which generally exhibit a lower GC content relative to genes associated with photosynthesis and the genetic system (Jansen and Ruhlman, 2012). Notably, these chloroplast genomes are considerably larger than the average chloroplast genome size of 150 kb typically observed in most flowering plants (Ruhlman and Jansen, 2014). Although the IR region is often regarded as the most conserved segment of the chloroplast genomes, variations in its boundaries, resulting from contraction and expansion, are commonly observed throughout chloroplast genome evolution. These variations are associated with changes in the length of the chloroplast genomes and may lead to phenomena such as gene loss, gene duplication, gene rearrangement, and pseudogenization in certain angiosperms (Zhu et al., 2016). A notable structural change observed in *P. pubinerve* includes an inversion of the *ndh*I gene, alongside the absence of the *ndh*A gene. In this species, the *ndh*A gene was transferred from the SSC region to the IR region due to the expansion of the IR region, and the instability at the IR/SSC junction may be associated with the deletion of the *ndh*A gene. The *ndh*A gene is a member of the NADH dehydrogenase gene family. In the light reaction phase of photosynthesis, the protein encoded by this gene actively engages in the photosynthetic electron transport chain, promoting the progress of the light reaction and ensuring the highly efficient functioning of photosynthesis. Some studies have revealed that the hemiparasitic trait in plants can hasten the pseudogenization and loss of plastid NDH genes (Li et al., 2021). The exact reason for the absence of the *ndh*A gene in the chloroplast genomes of *P. pubinerve* remains challenging to explain. The species-specific loss of certain NDH genes across different taxa suggests that not all NDH genes are universally involved in or required for photosynthetic electron transport. The functional deficiency caused by the loss of a single NDH gene (e.g., *ndh*A) may be compensated by other NDH genes or nuclear-encoded genes (Mohanta et al., 2020). Therefore, whether the absence of *ndh*A in *P. pubinerve* is linked to its ecological environment in the shaded and wet forest deserves further study. Inversions within plastomes are recognized as a powerful phylogenetic feature due to their rarity and minimal homoplasy (Schwarz et al., 2015). However, underlying mechanisms driving inversions in plant genomes remain poorly understood. In the chloroplast genomes of *P. hainanense*, *P. yunnanense*, *P. tonkinense* and *S. placentarium*, as the IR regions expands towards the SSC regions, the *ycf*1 gene, which spans the SSC/IRa boundary, exhibits partial duplication at the IRb/SSC boundary. This truncation likely results in a nonfunctional protein, and thus *ycf*1 is considered as a pseudogene. At the transcriptional level, duplicated parts may alter the chromatin structure around the *ycf*1 gene, interfering with the transcription factors’ binding to the promoter. This reduces *ycf*1 transcription and mRNA abundance, ultimately impacting protein expression levels. At the protein level, when duplicated parts are located near the crucial domains or functional sites of the encoded protein, they disrupt its folding, resulting in an abnormal structure that impedes related function. The *ycf*1 pseudogene could serve as a novel molecular marker, providing additional phylogenetic insights. By comparing the presence/absence of the *ycf*1 pseudogene, sequence characteristics, and IR region variations across species, it is possible to reconstruct plant evolutionary relationships and reveal their history (Dong et al., 2015). While pseudogenes typically evolve under neutral selection, some research indicates that they can be repurposed as functional genes with distinct roles following intense purifying selection pressure (Mahmudi et al., 2015). It is noteworthy that the expansion of IR regions appears to occur more progressively in monocots compared to non-monocot angiosperms, with a greater number of LSC genes being converted into IRs (Wang et al., 2008).

### 4.2 Simple sequence repeats and codon usage analysis

SSRs were extensively distributed in the chloroplast genomes of eukaryotic organisms. Due to their simple structure, relative conservation, and polymorphism, SSRs are recognized as effective molecular markers and are widely used in species identification, genetic diversity, and population genetics research (Powell et al., 1995; Provan et al., 2001; He et al., 2012). In the chloroplast genomes of *Phrynium* and *Stachyphrynium*, a total of 73 to 119 SSRs were identified, encompassing repeat units ranging from mononucleotides to hexanucleotides. The findings for both genera align with the typical characteristics of SSRs observed in the chloroplast genomes of angiosperms, particularly the predominance of mononucleotide A/T repeats, which are generally composed of short polyadenine (poly A) or polythymine (poly T) repeats, while G or C repeats are infrequently encountered (Powell et al., 1996). Mononucleotide nucleotide repeats may play a more significant role in genetic variation compared to other types of SSRs. Furthermore, SSRs are primarily located in the LSC region, indicating a high level of nucleotide variability in this area, which may provide valuable insights for detecting both intraspecific and interspecific polymorphisms at the population level.

Throughout the course of long-term evolution, species have gradually evolved specific codon usage patterns during the process of genes encoding proteins in response to their physiological requirements. This phenomenon is referred to as codon usage bias (Zhou et al., 2008; Sheng et al., 2021). RSCU is a critical metric for examining codon usage bias. In *Phrynium* and *Stachyphrynium*, the number of high-frequency codons with an RSCU greater than 1 is 29 and 28, respectively, with the majority end with an A/U base. This observation indicates a strong preference for A/U-ending codons in these genomes, a trend that is consistent with findings in other angiosperms (Nanjala et al., 2022; Jiang et al., 2023; Xu et al., 2023). Codons are essential for the accurate expression of genetic information, and codon usage bias is closely linked to the long evolutionary history of organisms. Consequently, investigating codon utilization in these genera is of considerable significance for the exploration of the evolutionary models of species in *Phrynium* and *Stachyphrynium*.

### 4.3 Molecular markers

Highly variable genomic regions are widely used as DNA barcoding markers for species identification and phylogenetic studies. The identification of species in *Phrynium* and *Stachyphrynium* presents challenges due to the pronounced morphological similarities among them. Given that certain traditional DNA barcode fragments are inadequate for accurate species identification and phylogenetic analysis within these genera, it is essential to explore more highly variable regions at the generic level that may serve as effective markers for future studies on species differentiation. Through mVISTA and nucleotide diversity analyses, four highly variable intergenic regions (*trn*S-*trn*G, *atp*H-*atp*I, *trn*E-*trn*T, *trn*T-*trn*L) were identified in seven *Phrynium* species and two *Stachyphrynium* species. Furthermore, the coding regions *ycf*1 and *ycf*2 exhibited significantly higher variability in *Phrynium*. The *ycf*1 gene has been recognized as the most variable region in the chloroplast genomes, exhibiting a mutation rate that exceeds that of established chloroplast barcode candidates such as *ma*tK and *rbc*L. This has led to its increasing application as a potentially valuable molecular marker, particularly in recent studies of orchids (Neubig et al., 2008; Whitten et al., 2014; Dong et al., 2015). Additionally, *ycf*2 alone can provide a well-supported phylogeny that generally corresponds with phylogenetic trees derived from more comprehensive multigene or plastid genomes datasets (Huang et al., 2010). The intergenic regions identified in the current study have also been documented in other plant species at the species level. For instance, the *atp*H-*atp*I and *trn*S-*trn*G regions exhibited significant variability, facilitating effective species differentiation in Aroideae subfamily (Araceae) (Dong et al., 2012; Li et al., 2022). The *trn*T-*trn*L region has been employed to analyze the phylogenetic relationships of *Kengyilia* (Poaceae) species (Gao et al., 2014).

Phylogenetic trees of seven species of *Phrynium* and two species of *Stachyphrynium* utilizing four molecular markers that were independently and combinatorially screened were also contructed. The resulting trees exhibited a topology consistent with that of the CDS and the nrDNA tree, revealing that *Phrynium* is further divided into two distinct clades, while *Stachyphrynium* constituted an independent clade. However, certain groups of species (such as *P. tonkinense*, *P. pedunculiferum*) within each tree were not clearly distinguished (**Supplementary Figures 2A–F**). These results suggest that the identified regions may function as potential molecular markers and offer significant resources for phylogenetic investigations at both interspecific and intraspecific levels. Although several candidate barcode regions have been recognized, further research is necessary to assess the effectiveness of these highly differentiated markers.

### 4.4 Phylogenetic relationships

Prince and Kress (2006) conducted phylogenetic analyses of Marantaceae using *mat*K, the 3’ intergenic spacer region, and the *trn*L-F region. Their findings indicated that *Phrynium* is non-monophyletic, with *Phacelophrynium* nested within it. The *Stachyphrynium* clade, which includes *Stachyphrynium*, was grouped with the *Maranta* clade and formed a sister clade to the Donax clade (which includes *Phrynium*). Suksathan et al. (2009) further analyzed phylogenetic relationships within Asian Marantaceae using the *rps*16 intron, ITS1 and NTS. The results revealed that *Stachyphrynium* constitutes a strongly supported monophyletic group, while the genera, *Phrynium*, *Phacelophrynium*, *Monophrynium* and *Cominsia* form a well-supported monophyletic clade. Based on these findings, they proposed consolidating these four genera within the *Phrynium* complex into a single genus, as phylogenetic evidence does not support their distinctiveness. Furthermore, *Stachyphrynium* exhibits closer phylogenetic relationship with African genera. Clearly, comprehensive taxon sampling of *Phrynium* is essential to evaluate its monophyly and clarify relationships with other genera.

In this analysis, *Phrynium* and *Stachyphrynium* are positioned on separate branches. *Phrynium* is further subdivided into two clades supported by morphological characteristics, while *Stachyphrynium* forms an independent clade. Within clade I, *P. pyramidale*, *P. imbricatum* and *P. pubinerve* clustered closely. Their synapomorphies include a shorter peduncle (<3 cm). Four species—*P. hainanense*, *P. tonkinense*, *P. pedunculiferum*, and *P. yunnanense*—are grouped into Clade II. Clade II is characterized by having subtriangular fruits and most of them have a long peduncle, except *P. tonkinense*. The shortened peduncle might be a consequence of the continuous adaptive evolution of plants in response to environmental pressures. *P. yunnanense* is distinguished from the other species by its longer peduncle (20-45 cm), bright orange bracts and fruits, along with green flowers. The remaining species exhibit clear distinctions: while both *P. pedunculiferum* and *P. hainanense* produce white fruits, *P. pedunculiferum* is characterized by green, leathery bracts that remain intact at maturity. In contrast, *P. hainanense* has bracts that dissolve into a blackish fibrous mass. Additionally, *P. tonkinense* is notable for its distinctly longer and narrower lanceolate leaves compared to other species in the genus.

It is a commonly observed that phylogenetic trees constructed from chloroplast and nuclear genomes frequently exhibit topological inconsistencies. These discrepancies may result from incomplete lineage sorting, horizontal gene transfer, recombination or convergent molecular evolution (Steenwyk et al., 2023). The phylogenetic position of *P. yunnanense* exemplifies this issue. However, it receives weak support in both genome-based trees, and as only one sample is included, its phylogenetic placement remains uncertain, warranting further investigation.

### 4.5 The new species

The new species, *Phrynium pyramidale*, exhibits a suite of unique morphological characteristics, including a pagoda-like inflorescence, reddish-brown fertile bracts that fragment into numerous fibers apically, yellow flowers, and subtriangular brown fruits. Its distinct species status is further supported by molecular evidence. Phylogenetic analyses of 40 samples of *Phrynium* and *Stachyphrynium*, based on chloroplast CDS and nrDNA datasets (**Figure 8**), reveal that eleven populations of *P. pyramidale* form a strongly supported monophyletic clade, which is sister to *P. pubinerve* and *P. imbricatum*. *P. pyramidale* is characterized by bracts arranged in a pagoda-like structure with the apex decaying rapidly but never exceeding one-third of the total length of bracts. Molecular data indicate that *P. imbricatum* and *P. pubinerve* are the closest relatives of *P. pyramidale*. However, *P. imbricatum* can be distinguished by its green, leathery larger bracts (2.8-3.7 × 1.3-2.4 cm), with entire apices and seeds that bearing a large appendages. In contrast, *P. pubinerve* is characterized by purplish-red flowers, bright red fruits and bracts that rapidly wither and dissolve into a blackish fibrous mass.

## 5 Conclusion

In this study, we conducted a comprehensive analysis of chloroplast genomes across *Phrynium* and *Stachyphrynium*, including genome assembly, comparative genomics, and phylogenetics. Results reveal strong structural conservation regarding structure, gene content, gene arrangement, and codon usage bias. However, divergences were detected in genome size and the SC/IR boundaries. We further identified genomic hotspot regions contributing to intergeneric differentiation, which could serve as potential DNA barcodes for species identification. Phylogenetic analysis based on CDS (coding sequences) and nrDNA (nuclear ribosomal DNA) strongly support the monophyly of *Phrynium* and *Stachyphrynium* with *Phrynium* further subdivided into two distinct clades. These findings deepen our understanding of their molecular evolution and provide critical insights for identification, utilization, and conservation of these medicinal plants. Additionally, we describe and illustrate a new species, *P. pyramidale*.

## 6 Taxonomy

**Figure 9:**
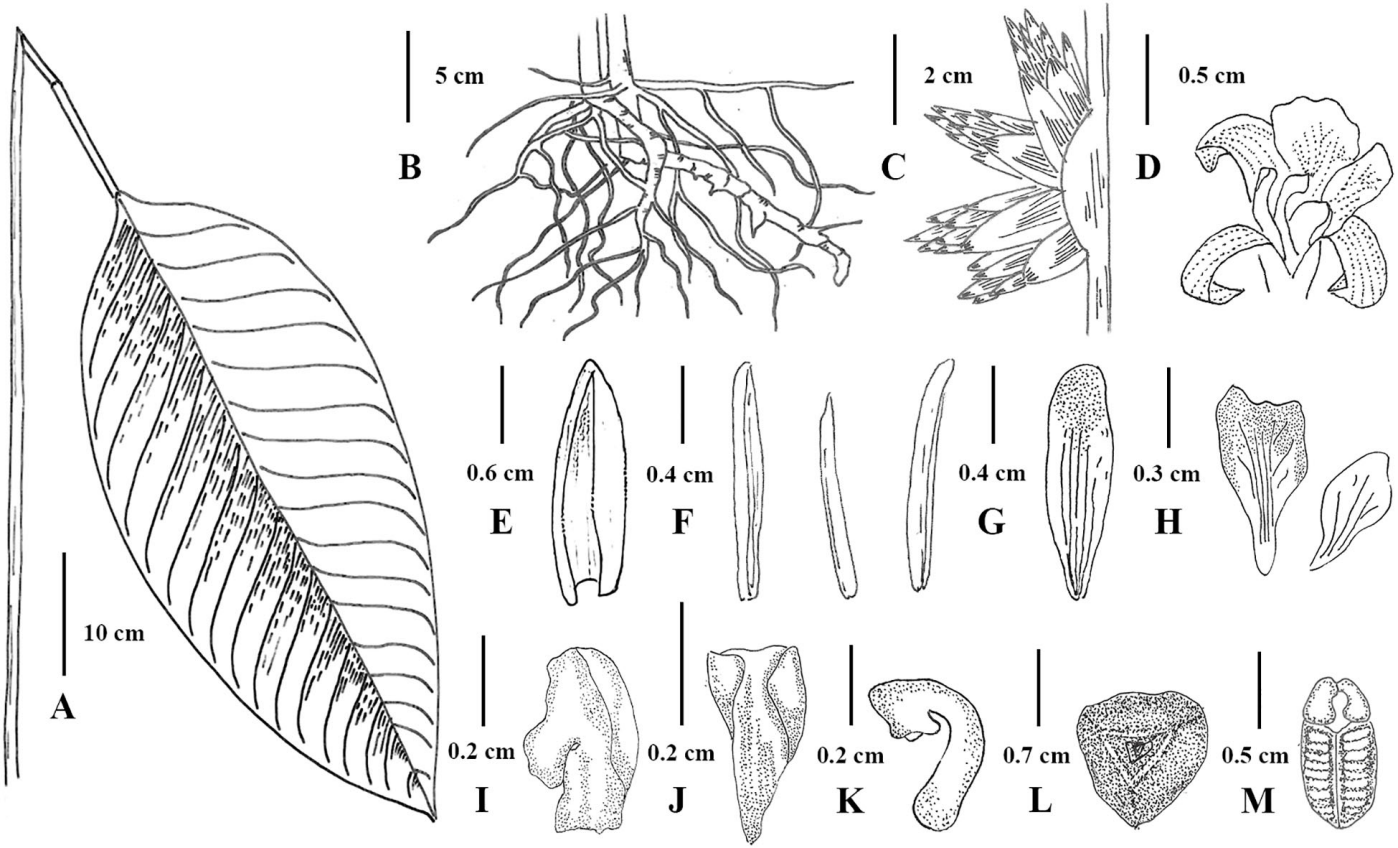
Illustration of *Phrynium pyramidale* **(A)** Leaf; **(B)** Leave sheaths and rhizome; **(C)** Inflorescence; **(D)** Flower; **(E)** Prophyll; **(F)** Sepals; **(G)** Petal lobe; **(H)** Outer staminodes **(I)** Callose staminode; **(J)** Cucullate staminode; **(K)** Stigma; **(L)** Top view of fruit; **(M)** Seed.

**Figure 10:**
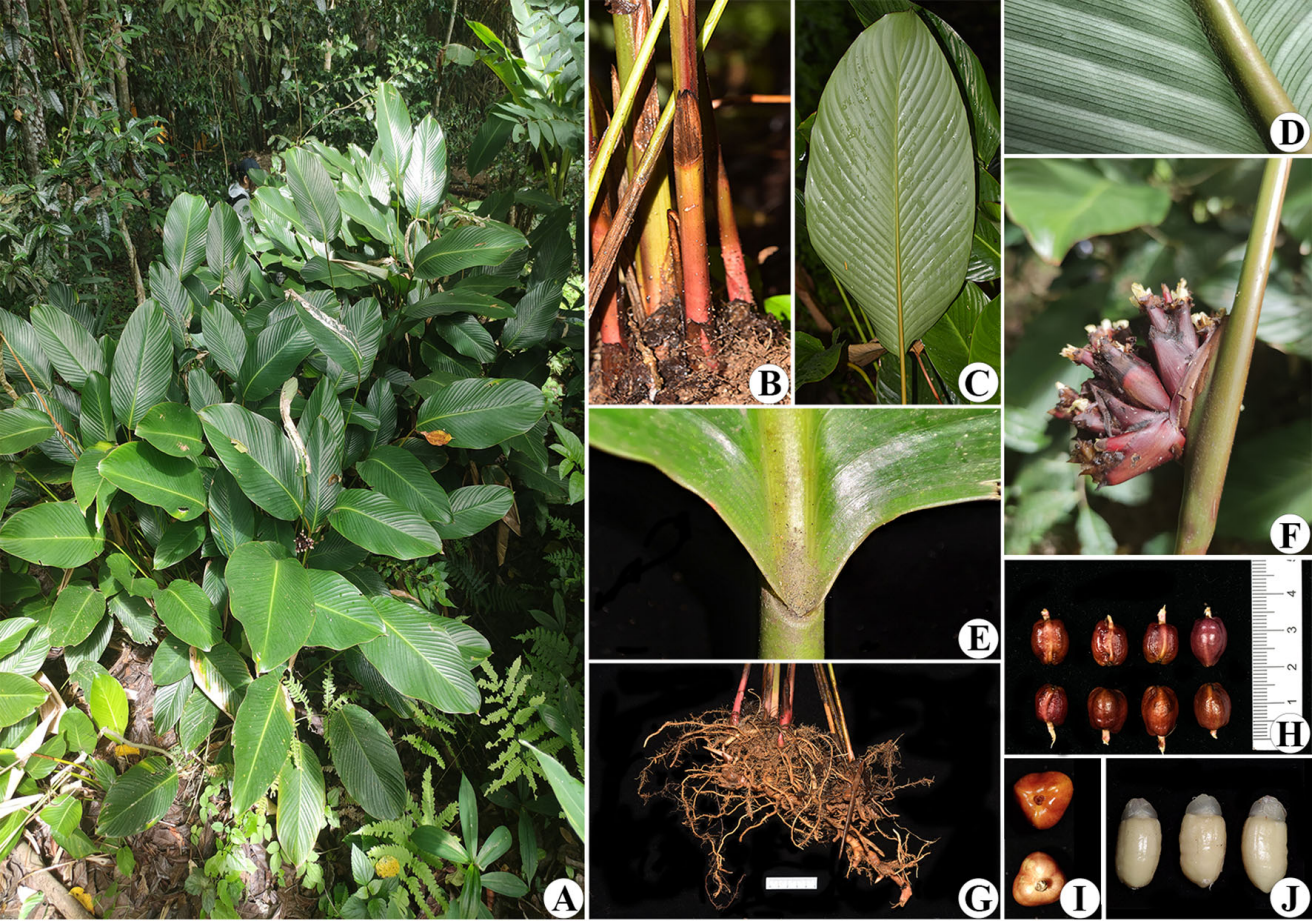
*Phrynium pyramidale* **(A)** Habit; **(B)** Leave sheaths; **(C)** Abaxial leaf surface; **(D)** Details of the abaxial leaf surface; **(E)** Lamina base and pulvinus; **(F)** Inflorescence with blooming flowers; **(G)** Rhizome; **(H, I)** Young fruits; **(J)** Seeds.

**Figure 11:**
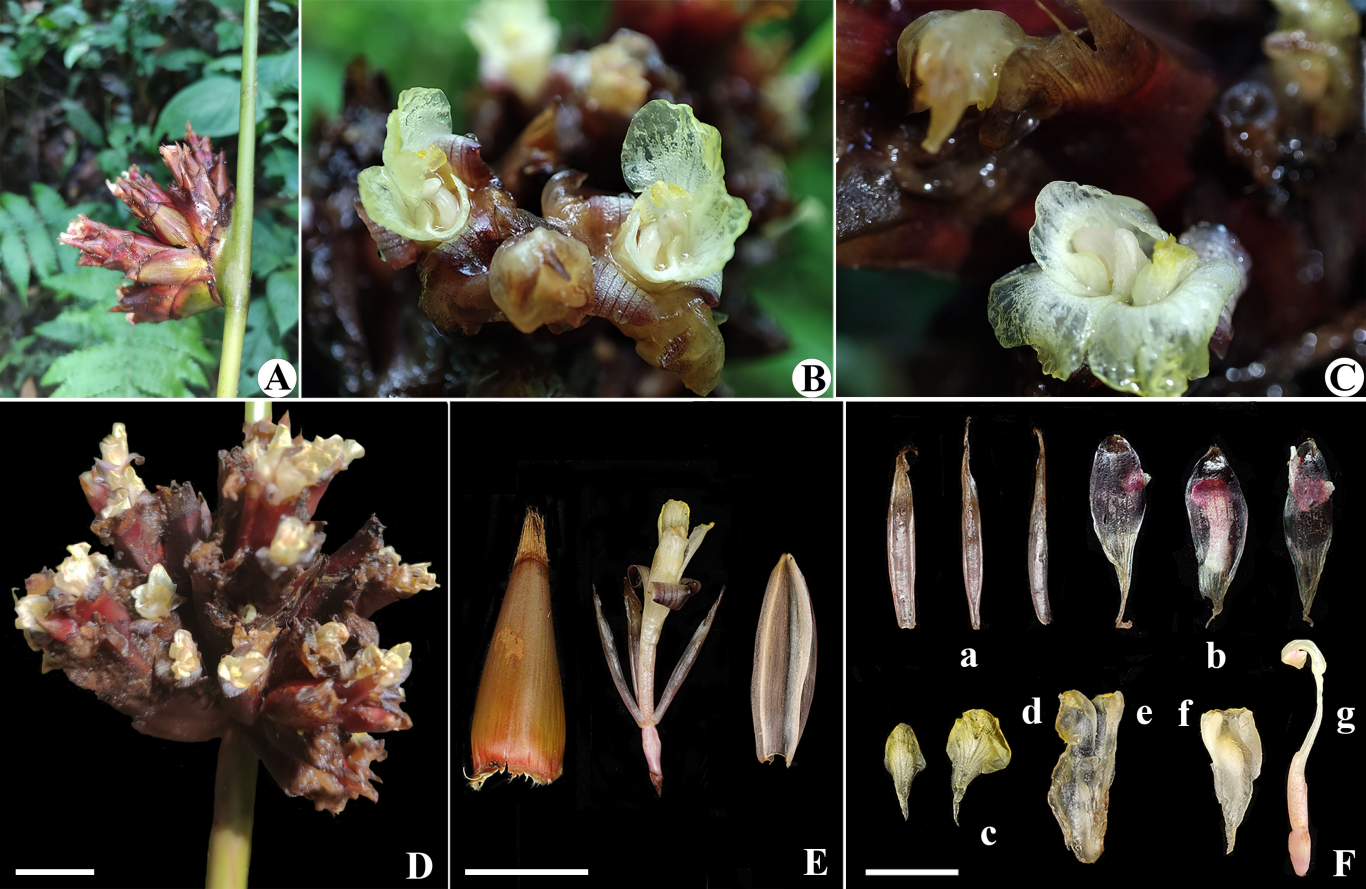
*Phrynium pyramidale* **(A)** Lateral view of inflorescence; **(B, C)** Close up of flower; **(D)** Top view of inflorescence; **(E)** Fertile bract, flower and prophyll; **(F)** Flower dissection: **(a)** Sepals; **(b)** Petal lobes; **(c)** Outer staminodes; **(d)** Callose staminode; **(e)** Fertile stamen; **(f)** Cucullate staminode; **(g)** Pistil. Scalebars=1cm.

**Figure 12:**
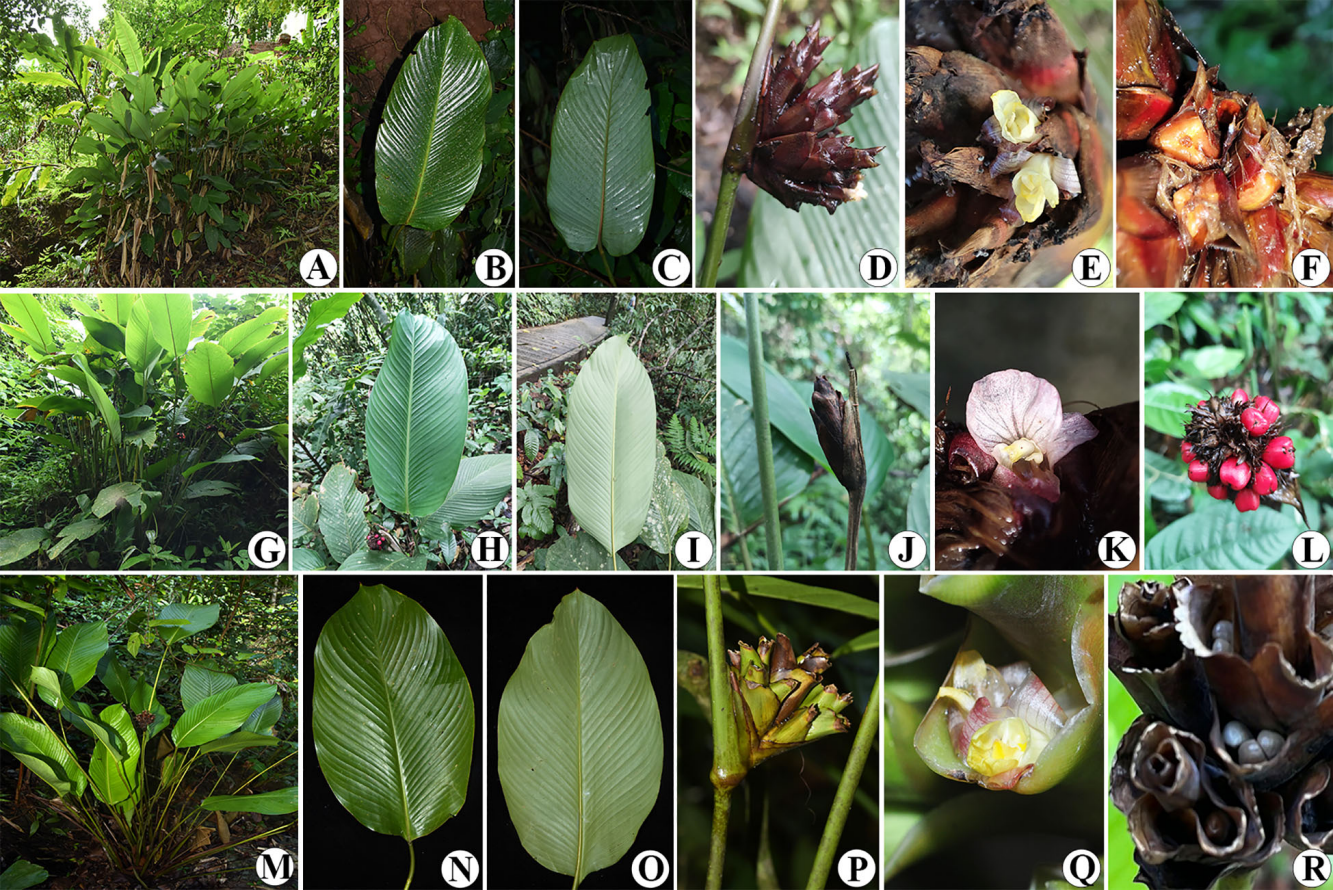
*Phrynium pyramidale* **(A)** Habit; **(B)** Adaxial leaf surface; **(C)** Abaxial leaf surface; **(D)** Inflorescence; **(E)** Close up of flower; **(F)** Young fruits. *Phrynium pubinerve* **(G)** Habit; **(H)** Adaxial leaf surface; **(I)** Abaxial leaf surface; **(J)** Inflorescence; **(K)** Close up of flower; **(L)** Young fruits. *Phrynium imbricatum* **(M)** Habit; **(N)** Adaxial leaf surface; **(O)** Abaxial leaf surface; **(P)** Inflorescence; **(Q)** Close up of flower; **(R)** Young fruits.

*Phrynium pyramidale* Y. Tong & Z. Y. Lin, sp. nov. (**Figure 9**–**12**)

Diagnosis:—*Phrynium pyramidale* is similar to *Phrynium pubinerve* and *Phrynium imbricatum*., but it is distinguished by having pagoda-like inflorescence, the reddish-brown fertile bracts which only splitting up into many fibers at the tip, the yellow flowers and the subtriangular brown fruits. (**Table 5**).

**Table 5:** Morphological comparison of *Phrynium pyramidale*, *P. pubinerve* and *P. imbricatum*.

Characters	*P. pyramidale*	*P. pubinerve*	*P. imbricatum*
Sheath	glabrous	densely pubescent	glabrous
Leaves	2-6 per shoot, abaxially fresh green, adaxially light green	2-4 per shoot, abaxially light green, adaxially yellowish green	2-3 per shoot, abaxially dark green, adaxially yellowish green
Pulvinus	6-12 cm long	2-12.5 cm long	3-12 cm long
Bracts	broadly elliptical, 2-2.9 × 1.6-2.2 cm, yellowish green	broadly ovate to narrowly elliptic, 1.9-6.7 × 1.1-3.7 cm, yellowish green or brown	ovate-oblong, 2.8-3.7 × 1.3-2.4 cm, reddish-brown or light green
Fertile bracts	reddish-brown, broadly lanceolate or broadly elliptical, glabrous	broadly elliptic, reddish-brown or yellowish green	ovate to ovate-lanceolate, reddish-brown or light green
Flowers	yellow, 2.2-2.5 cm long	purplish-red, 1.8-2.0 cm long	yellow, 2.5-2.8 cm long
Sepals	subulate, 11-13 × 2.5-3 mm	linear, 8.7-11 × 2.0-2.5 mm	linear, 10.2 × 1.1 mm
Petal lobes	elliptic to oblong, 11-13 × 3.5-4 mm, pale purple	oblanceolate, 6.2-8.8 × 2.5-3.1 mm, purplish-red	elliptic-oblong, 6-7.3 × 3 mm, purplish-red
Ovary	glabrous	densely hairy	glabrous
Fruits	reddish brown, elongate to triangular, 12-14 × 10-12 mm	bright red, elongate to triangular, 11-15 × 10-12 mm	light brown, elongate to triangular, 11-13 × 7-10 mm
Seeds	grey, trigonous, 9-11 × 5-7 mm, shallowly grooved	grey, subquadrangular, 9-11 × 6-7 mm, slightly rough, pubescent, with translucent, bifid appendages	white, slightly trigonous ellipsoid, 10-11 × 5-6 mm, shallowly grooved, with very large appendages, 3 mm in diam

Type:—CHINA. Yunnan Province: Xishuangbanna Dai Autonomous Prefecture, Xiding Country, Zhanglang Village, growing on a gentle slope situated beneath the forest adjacent to the temple, 21°54’58.78”N, 100°07’08.12”E, 1,542 m a.s.l., 8 July 2023, *Tong Yi TY230708014* (holotype: GUCM!, isotype: IBSC!).

Description:—Rhizomatous ground herb 0.6-2 m tall. Basal leaves 2-6 per shoot; sheath 20-55 cm long, red to brown, glabrous; petiole 40-135 cm long, light green, glabrous; pulvinus 6-12 cm long, yellowish green, glabrous; lamina oblong to elliptic, 40-55 × 13-27 **cm**, thinly leathery, adaxially fresh green, glabrous, abaxially light green, glabrous to sparsely hairy, base rounded, apex acuminate, acumen 0.5-1.5 cm. Inflorescence interfoliar, erect, peduncle largely obscured by an enveloping leaf sheath, 0.8-1.5 cm long, light green, glabrous; synflorescence capitate, densely congested, 5-8 cm in diameter; bracts subtending the proximal branches broadly elliptical, 2-2.9 × 1.6-2.2 cm, yellowish green, soon becoming dry and turning brown, broadly elliptical; fertile bracts 1.5-3.4 × 1.2-2.3 cm, reddish-brown, glabrous, broadly lanceolate or broadly elliptical, spirally arranged, apex initially acute, soon splitting up into many fibres at the tip; flower pairs 3 per special paraclade, associated prophylls 17-20 × 8.5-10 mm, ovate to lanceolate, with two distinct keels on the abaxial surface and incurved at margins, interphylls 14-18 × 4 mm. Flowers 2.2-2.5 cm long; sepals 3, subulate, 11-13 × 2.5-3 mm, yellowish-white, semi-translucent; corolla tube 8 mm, cylindric; staminodial tube as long as the corolla tube; petal lobes elliptic to oblong, 11-13 × 3.5-4 mm, semi-translucent pale purple, dotten with small purple spots, deflexed and curled; outer staminodes 2, slightly unequal, petaloid lobes broadly obovate, 5-7 × 2.5-3.5 mm, light yellow; cucullate staminode 3-3.5 × 1.5-2 mm, cream-white, yellow at the tip; callose staminode 3.5-4 × 2.5-3 mm; fertile stamen small, 3 mm long; style with free part 6 mm long, curved inwards, cream-white; stigma light yellow, ca. 2 mm in diameter; ovary oblong, 3-4 mm long, 3-loculed. Fruits reddish brown, shiny, elongate to triangular, 12-14 × 10-12 mm; seeds 3, grey, trigonous, shallowly grooved, 9-11 × 5-7 mm, arillate, aril cream-white, extending into 2 curled subulate, translucent appendages.

Etymology:—The specific epithet refers to the pagoda-like inflorescence.

**Figure 13:**
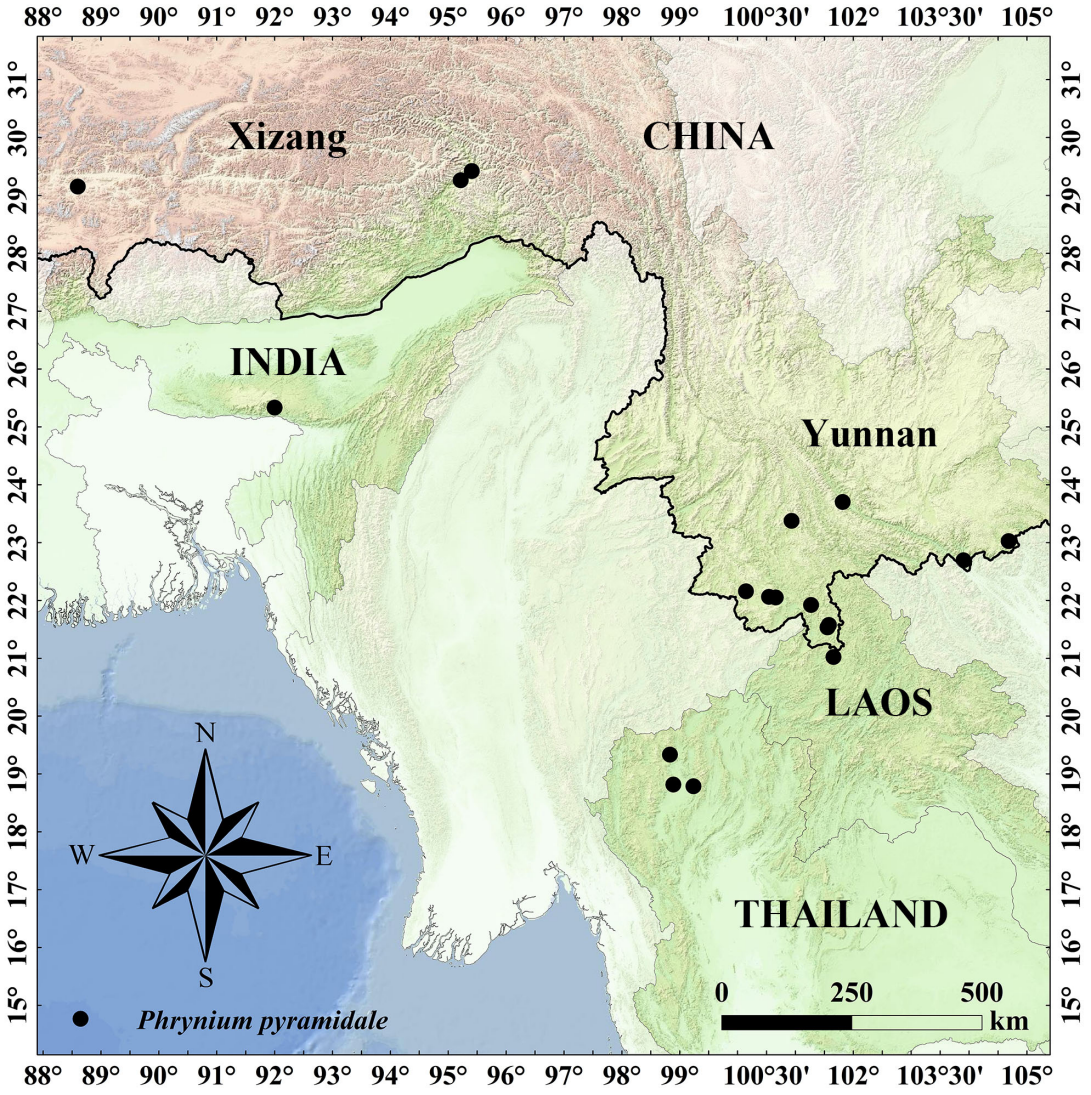
Distribution map of *Phrynium pyramidale* (black circle).

Habitat, distribution and phenology:—*Phrynium pyramidale* is currently known from Yunnan and Xizang. And it also found in Laos, India and Thailand (**Figure 13**). It inhabits in moist valleys and dense forests, at an altitude of about 1,000-1,500 m. Flowering from June to August; fruiting from July to September.

Conservation status:—*Phrynium pyramidale* is widespread in China, south to Laos and Vietnam, and southwest to India. It exhibits well-developed vegetative reproduction, and is also highly adaptable to the local natural environment and capable of surviving in human-disturbed habitats. It is commonly cultivated by local communities around homes for use in wrapping traditional rice dumplings during festivals. Its populations are often large and frequently encountered within its distribution range. Therefore, according to the IUCN Red List Categories and Criteria (IUCN, 2024), it should be assessed as Least Concern (LC).

Specimens Examined:—CHINA: Yunnan: Menghai, Mengsong Township, Sanmai Village, 22°03’00”N, 100°39’34”E, 1,541 m a.s.l., 24 Apr. 2012, *Menghai Census Team 5328220074* (IMDY); Yuanjiang, Donge Town, Namujing, 23°42’N, 101°49’E, 1,000 m a.s.l., 28 May. 1983 (fl.), *Tao G.D. 38659* (HITBC, KUN); Puer, Jinggu, Zhengxing town, 23°22’29.17”N, 100°55’58.71”E, 981 m a.s.l., 17 Dec. 2019, *ZXX191871* (KUN); Wenshan, Maguan, Gulinjing County, Macaoping Village, 22°41’35.83”N, 103°54’22.66”E, 950 m a.s.l., 12 May. 2019 (fl.), *Z. L. Dao, L. Cai, P. Zhang KIBDZL168B09* (KUN); 1,450 m a.s.l., 29 May. 2013 (fl.), *L. Wu 3935* (BNU); Lincang, Mayidui Village, Near Nanbo River, 1,350 m a.s.l., 24 Aug. 1957 (fr.), *J. S. Xin 333* (IBSC, KUN, LBG, PE); Xishuangbanna, Jinghong, Mengyang Town, Kunluo Highway, 1400 m a.s.l., 7 Jul. 1957 (fr.), *Sino-Soviet Joint Expedition 5956* (IBSC, IBK, KUN). Xizang: Linzhi, Motuo, Miri Village, 29°25’18.04”N, 95°24’26.30”E, 833 m a.s.l., 27 May. 2013 (fl.), *C. Liu, J. Cai, T. Zhang 13CS6428* (KUN); From Tea garden to Buqunhu Lake, 29°15’37.75”N, 95°12’57.20”E, 1,101 m a.s.l., 14 Nov. 2016, *C. Liu, J. D. Ya, H. J. He 16CS11896* (KUN). LAOS: Oudomxai, Ban Nam Pheng, 21°01’12”N, 101°39’25”E, 800 m a.s.l., 23 Jun. 1999, *M. F. Newman 875* (E). INDIA: Assam, Nongpoh, Khasi Hills, 2,000 ft., 13 May. 1949, *Koelz WN 22692* (L); Meghalaya, Cherrapunjee, Khasi Hills, 4,000 ft., 15 May. 1952. *Thakur Rup Chand 5647* (MICH). THAILAND: Chiengmai: Doi Sutep, 18°48’58.5”N, 98°53’30.52”E, 1,120 m a.s.l., 17 Apr. 1958, *Sørensen, T; Larsen, K; Hansen, B 2838* (L); Ru-See Cave area, 18°47’14.68”N, 99°14’17.23”E, 1,075 m a.s.l., 10 Oct. 1987, *Maxwell, JF 87-1148* (L); Chang Kian Valley, 18°48’58.5”N, 98°53’30.52”E, 1,000 m a.s.l., *Maxwell, JF 88-619* (L); Chieng Dao base hill, 19°20’N, 98°50’E, 600-700 m a.s.l., 6 May. 1973, *R. Geesink 5723* (AAU, L, P).

## Data Availability

The original contributions presented in this research are accessible to the public. The data that support the findings of this study can be found in the Genbank database at https://www.ncbi.nlm.nih.gov/under the accession number PQ835413-PQ835452, PQ865401, PQ865465, PQ865467, PQ865470-PQ865471, PQ865473-PQ865474, PQ865476-PQ865486, PQ865488-PQ865492, PQ865494, PQ869814-PQ869829.
