# Supplementary material for: Comparative and phylogenetic analysis of complete chloroplast genomes of Phrynium s. s. and Stachyphrynium (Marantaceae) in China, including a new species
Source: Front Plant Sci. 2025 May 1;16:1569683. doi: 10.3389/fpls.2025.1569683 (PMC12078270; doi:10.3389/fpls.2025.1569683)
Supplement: Supplementary file 1 [file DataSheet1.docx]

Supplementary Material

## Supplementary Figures

##
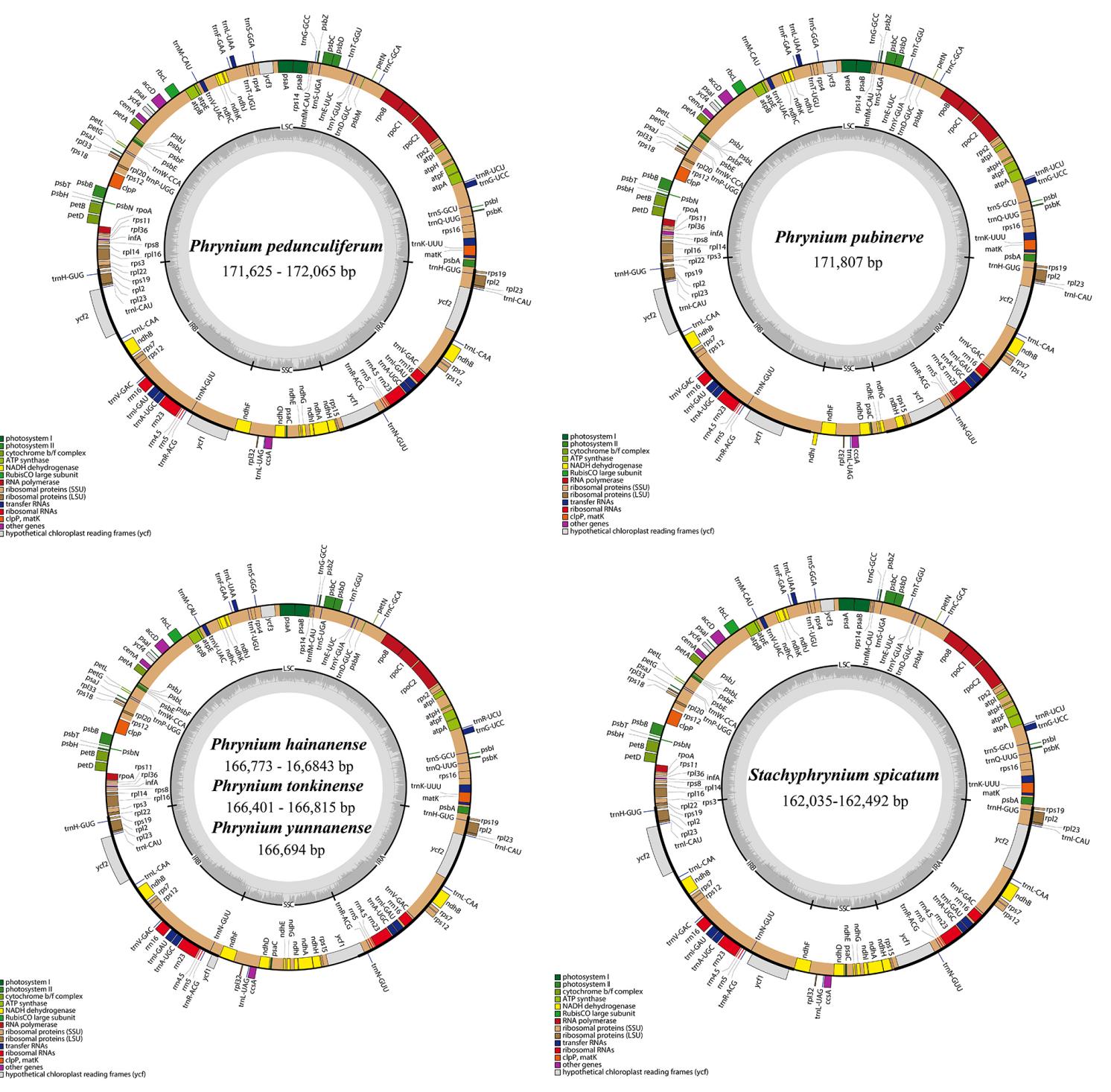
**Supplementary Figure 1.** The complete chloroplast genome maps of two *Phrynium* and one *Stachyphrynium* species.


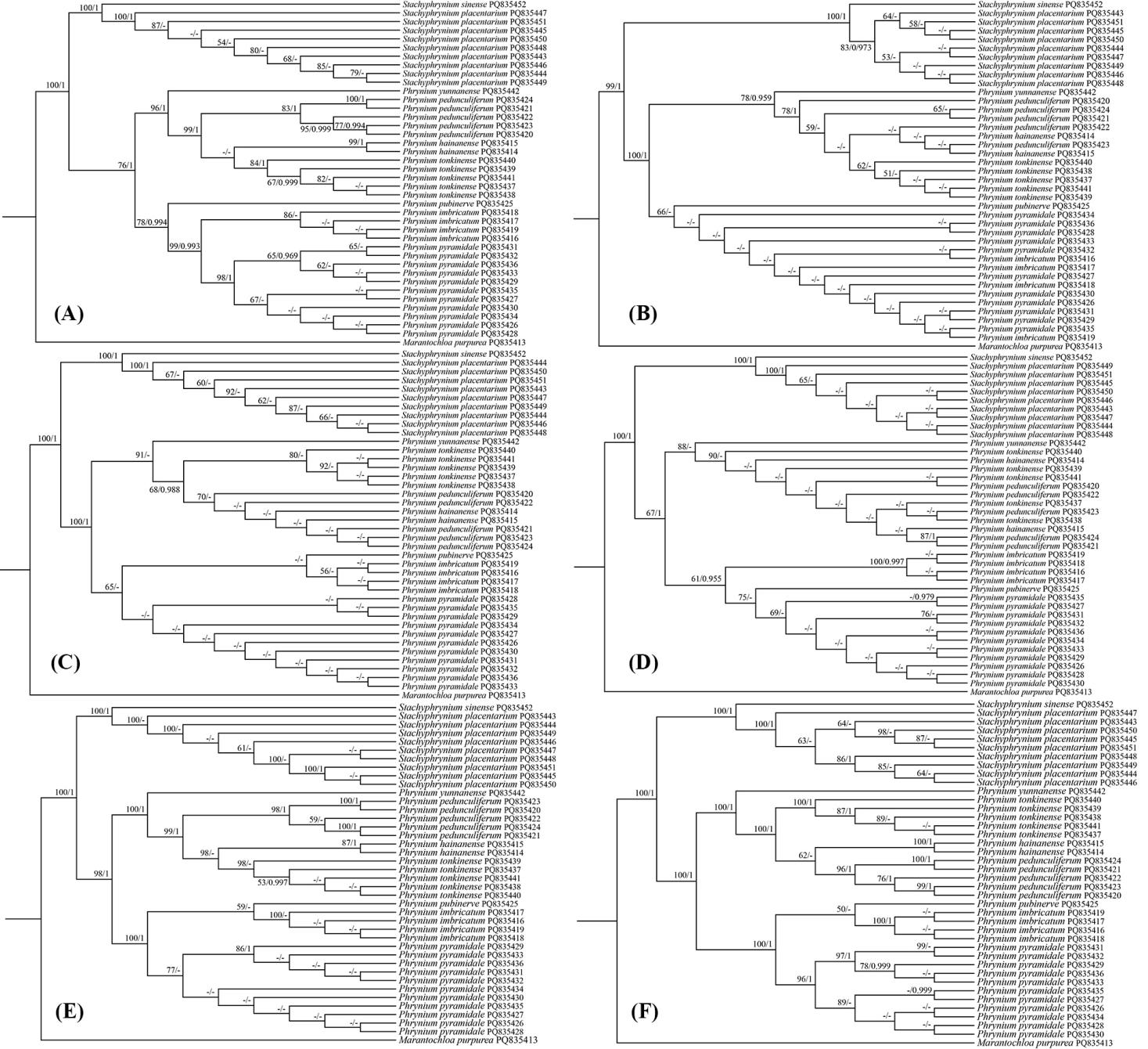


**Supplementary Figure 2.** Phylogenetic trees based on the highly-variable regions in chloroplast genome. (A) *trn*S-*trn*G; (B) *atp*H-*atp*I; (C) *trn*E-*trn*T; (D) *trn*T-*trn*L; (E) *ycf*1; (F) combined analysis of sequences *trn*S-*trn*G, *atp*H-*atp*I, *trn*E-*trn*T, *trn*T-*trn*L and *ycf*1.
